# How Can Inequalities in Mortality Be Reduced? A Quantitative Analysis of 6 Risk Factors in 21 European Populations

**DOI:** 10.1371/journal.pone.0110952

**Published:** 2014-11-04

**Authors:** Terje A. Eikemo, Rasmus Hoffmann, Margarete C. Kulik, Ivana Kulhánová, Marlen Toch-Marquardt, Gwenn Menvielle, Caspar Looman, Domantas Jasilionis, Pekka Martikainen, Olle Lundberg, Johan P. Mackenbach, for the EURO-GBD-SE consortium

**Affiliations:** 1 Department of Public Health, Erasmus Medical Center, Rotterdam, the Netherlands; 2 Department of Sociology and Political Science, Norwegian University of Science and Technology (NTNU), Trondheim, Norway; 3 INSERM, Pierre Louis Institute of Epidemiology and Public Health, Paris, France; 4 Sorbonne Universités, Pierre Louis Institute of Epidemiology and Public Health, Paris, France; 5 Max-Planck-Institute of Demographic Research, Rostock, Germany; 6 Department of Sociology, University of Helsinki, Helsinki, Finland; 7 CHESS, Stockholm University/Karolinska Institutet, Stockholm, Sweden; Universität Bochum, Germany

## Abstract

**Background:**

Socioeconomic inequalities in mortality are one of the greatest challenges for health policy in all European countries, but the potential for reducing these inequalities is unclear. We therefore quantified the impact of equalizing the distribution of six risk factors for mortality: smoking, overweight, lack of physical exercise, lack of social participation, low income, and economic inactivity.

**Methods:**

We collected and harmonized data on mortality and risk factors by educational level for 21 European populations in the early 2000s. The impact of the risk factors on mortality in each educational group was determined using Population Attributable Fractions. We estimated the impact on inequalities in mortality of two scenarios: a theoretical *upward levelling scenario* in which inequalities in the risk factor were completely eliminated, and a more realistic *best practice scenario*, in which inequalities in the risk factor were reduced to those seen in the country with the smallest inequalities for that risk factor.

**Findings:**

In general, upward levelling of inequalities in smoking, low income and economic inactivity hold the greatest potential for reducing inequalities in mortality. While the importance of low income is similar across Europe, smoking is more important in the North and East, and overweight in the South. On the basis of best practice scenarios the potential for reducing inequalities in mortality is often smaller, but still substantial in many countries for smoking and physical inactivity.

**Interpretation:**

Theoretically, there is a great potential for reducing inequalities in mortality in most European countries, for example by equity-oriented tobacco control policies, income redistribution and employment policies. Although it is necessary to achieve substantial degrees of upward levelling to make a notable difference for inequalities in mortality, the existence of best practice countries with more favourable distributions for some of these risk factors suggests that this is feasible.

## Introduction

Inequalities in health between socioeconomic groups are increasingly recognized as one of the main challenges for health policy [1]. Studies comparing different European countries have shown that health inequalities are substantial almost everywhere, but that there are important variations between countries in the magnitude of health inequalities, suggesting great scope for reduction [2], [3].

Explanatory research has identified many factors contributing to inequalities in health. These include behavioural risk factors such as tobacco smoking, excessive alcohol consumption, and physical inactivity, but more ‘upstream’ social and economic risk factors such as social isolation, low income, unemployment, and occupational risks have been shown to contribute as well [4]–[8].

On the basis of these explanatory findings, policy proposals have been developed, both at the national level [9]–[14] and internationally (1). However, it is unclear what the potential for reducing health inequalities is in quantitative terms, and most of these proposals have not been based on a formal analysis of what the most important determinants of health inequalities are.

We have therefore set out to quantify the potential for reducing inequalities in mortality by tackling each of a number of key determinants. We have done this by estimating the reduction of educational inequalities in mortality that would be obtained, if European populations would succeed in reducing or even eliminating inequalities in important risk factors through effective policy interventions.

The first group of determinants is a set of three ‘downstream’ risk factors: *smoking*, *overweight*, and *physical inactivity*. These risk factors are relatively easy to measure and have a reasonably well-documented causal effect on mortality [15]–[18]. It has already been shown that these risk factors have a higher prevalence in lower socioeconomic groups in many European countries [19]–[23].

Because an emphasis on behavioural risk factors may distract from the necessary action on ‘upstream’ risk factors [24]–[26], we have also studied three social and economic risk factors: *lack of social participation*, *low income*, and *economic inactivity*. Lack of social participation, low income and economic inactivity (through temporary unemployment or more permanent detachment from the labour market) probably all increase mortality [27]–[34], and their prevalence is higher in lower socioeconomic groups [35]–[37].

The main purpose of this paper is to show to what extent inequalities in mortality can potentially be reduced by tackling each of these determinants, and to provide guidance on priorities for health policy in different European countries. As will be demonstrated, the potential for reducing inequalities in mortality is substantial, but priorities for action should not be the same everywhere.

## Data and Methods

### Data

Data on mortality by sex, age, cause of death and education were obtained from mortality registries for all European populations for which good quality data are available. They cover people aged 30–79 in 21 European populations in the period ca. 2000 – ca. 2005 and are mainly based on longitudinal or linked cross-sectional studies representing whole nations. These populations are those of Finland, Sweden, Norway and Denmark in the North; Scotland, England and Wales, Netherlands, Belgium, France, Switzerland, and Austria in the West; Spain (Barcelona, Basque Country and Madrid) and Italy (Turin and Tuscany) in the South, and Czech Republic, Poland, Hungary, Lithuania and Estonia in the Centre/East. Our main sources of mortality data are presented as supporting information (Table S1 in File S1).

We have focused on educational inequalities in mortality (instead of, e.g., occupational inequalities in mortality) because data on educational attainment are available for both men and women in all European populations under study. In addition, education is the most stable measure of socioeconomic position because it is normally completed early in adulthood, which avoids reverse causation problems (i.e., health problems at older ages cannot change a person's level of education) [38]. Educational level was harmonized across countries according to the International Standard Classification of Education (ISCED) and split into three internationally comparable categories. These correspond to less than secondary education (low), complete secondary education (mid), and tertiary education (high).

Risk factor prevalence data by sex, age, and level of education were collected for the early 2000s, mainly from national health surveys. Smoking was measured in three categories (‘never smokers’, ‘former smokers’, ‘current smokers’), overweight in three (BMI <25, 25–30, 30+), leisure-time physical activity in two (‘active’: less than once a week and ‘sedentary’: once a week or more), social participation in two (‘participation in at least one voluntary organization’, ‘no participation’), income in four (equivalent net household income quartiles), and economic activity in two (‘economically active’, ‘economically inactive’). Temporary unemployed were classified with the inactive in Norway, Sweden, Switzerland, and Madrid, and with the active in all other populations.

Relative risks for the impact of smoking, overweight, physical inactivity and lack of social participation on mortality were collected from large reviews and meta-analyses [18], [29], [39], [40] making sure that the estimates of relative risk were adjusted for the effect of relevant confounders. For income and economic inactivity no authoritative estimates were available in the literature. We therefore calculated relative risks for lower income quartiles using a Finnish register-based follow-up study of three million men and women, with adjustment for age, household structure, spouse's economic activity, occupational class, education and own economic activity [41]. We calculated relative risks for economic inactivity from our own mortality data, adjusting for age and educational attainment, and estimating a separate relative risk for countries where unemployed were classified with the inactive. A full account of all data sources can be found elsewhere [42]. All data used in the calculations and their definitions are presented as supporting information. These include characteristics of the mortality data (Table S1 in File S1), rate ratios for the association between education and all-cause mortality (Table S2 in File S1), relative risks for the impact of risk factors on all-cause mortality (Table S3 in File S1), sources of prevalences (Table S4 in File S1), and the prevalences of smoking (Table S5 in File S1), overweight (Table S6 in File S1), physical inactivity (Table S7 in File S1), social participation (Table S8 in File S1), income (Table S9 in File S1), economic inactivity (Table S10 in File S1). Furthermore, analyses of the potential reduction (in %) of relative educational inequalities in all-cause mortality between low and high educated, by risk factor, country and sex are given in Table S11 in File S1 (upward levelling scenario) and Table S12 in File S1 (best practice scenario).

Written informed consent of the usage of mortality and survey data was given by the relevant administrative units in the respective countries.

### Methods

We quantified educational inequalities in mortality by calculating rate ratios (RR) and rate differences (RD) from age-adjusted mortality rates using high education as a reference category. In this paper we only present scenario changes in RRs and RDs comparing the lowest with the highest educational group. Results for all educational groups can be found elsewhere [42].

The estimates of the potential reduction of inequalities in mortality are based on two different types of counterfactual scenarios. The first is an *upward levelling scenario* which assumes that the exposure to a risk factor would be reduced to the level currently seen among the highest educated within each country. This will identify a possible upper limit to what can be achieved in each country. In the uncommon case that the lower educated are less frequently exposed to risk factors than the higher educated, which mainly applies to female smokers in the South of Europe, we assume that the potential reduction of inequalities in mortality is zero.

The second type is *a best practice scenario*, in which we choose the country with the smallest educational inequalities in a risk factor (as identified by the upward levelling scenario, but making sure that small inequalities in this country stem from a low risk factor exposure among the low educated and not from a high risk factor exposure among the high educated). In the uncommon case that the prevalence of the risk factor was less favourable among high educated in the best practice country than in the country analysed, which mainly applies to smoking, we assume that the potential reduction of inequalities in mortality is zero. After we had identified the best-practice countries for each risk factor, we took the prevalences from both the highest and the lowest educational groups from these countries and applied them to other countries.

We used a specially developed, excel-based tool to quantify the expected changes in mortality that would result from modifying the population distribution of exposure to a risk factor. This tool is based on Population Attributable Fractions (PAF) and estimates the impact of counterfactual distributions of the risk factors on the magnitude of social inequalities in mortality [43].

The PAF is defined as the fraction of deaths which would have been avoided if the prevalence of a specific risk factor had been lower, and is measured with the following formula:
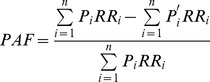



*n* =  number of exposure categories

*P_i_* =  proportion of population currently in the ith exposure category

*P*′*_i_* =  proportion of population in the ith exposure category in the counterfactual (alternative) scenario

*RR_i_* =  relative mortality risk for the ith exposure category

For the low- and mid-educated we first calculated age-specific PAFs in order to estimate new mortality rates and numbers of saved deaths in each age group (30–44, 45–59, 60–69 and 70–79). In a second step we summed up the age-specific saved deaths for the ages 30–79 years and calculated the overall PAF in each educational group.

To account for sampling variability, particularly of the risk factor distributions which were derived from survey data with limited sample sizes, we calculated 95% confidence intervals (CIs) around the PAF values of the lower educated using bootstrapping in R.

## Results

### Population-Attributable Fractions

Table 1 presents the educational inequalities in mortality as they were observed in the populations under study, on the basis of the mortality rate difference between the low and high educated, and shows that inequalities exist everywhere but vary importantly in magnitude: they are smallest in the South, and largest in the Centre/East.

**Table 1:** Mortality rate difference between low and high educated (in deaths per 100,000), by country and sex.

	Men					Women				
	Age group					Age group				
Population	30–44	45–59	60–69	70–79	**30–79**	30–44	45–59	60–69	70–79	**30–79**
Finland	291	537	926	1723	**615**	223	326	834	2188	**260**
Sweden	139	308	693	1539	**418**	76	203	431	930	**258**
Norway	198	438	989	2000	**579**	93	256	479	1069	**304**
Denmark	214	495	862	1660	**554**	103	259	590	970	**319**
England/W	75	321	662	1723	**410**	52	95	516	1167	**245**
Scotland	192	434	885	2112	**568**	120	240	623	1442	**366**
Netherlands	62	272	627	2248	**430**	49	134	382	609	**185**
Belgium	140	347	776	1646	**456**	61	131	304	871	**200**
France	201	507	902	2091	**599**	90	149	221	456	**166**
Switzerland	186	421	904	1720	**528**	72	114	188	666	**160**
Austria	168	478	958	1784	**557**	62	146	214	893	**193**
Barcelona	178	290	452	851	**325**	74	42	102	413	**98**
Basque C.	157	251	321	637	**262**	60	52	−22	251	**61**
Madrid	176	285	418	804	**312**	47	10	160	470	**90**
Turin	105	198	648	944	**304**	41	65	34	70	**51**
Tuscany	117	248	506	1011	**309**	47	64	162	243	**90**
Hungary	678	2140	2481	3801	**1788**	211	463	587	883	**425**
Czech R	207	868	2226	4388	**1158**	74	288	745	1970	**434**
Poland	623	1208	1971	3414	**1310**	180	293	643	1458	**413**
Lithuania	668	1531	2018	2719	**1389**	294	575	646	1199	**536**
Estonia	1039	1680	2423	3028	**1679**	451	657	858	1532	**691**
All ^#^										

# Median value of all populations included in the analysis.

Table 2 presents the PAF values for each risk factor for the low educated. Values range between a little above 0 (e.g., for physical inactivity among Czech men, and for overweight among Estonian men) to more than 10% (e.g. for smoking among men and women in several countries, and for low income among men in Scotland, Hungary and Poland), implying that between 0 and more than 10% of all deaths among the low educated could be avoided if they would have the risk factor prevalence of the high educated in the same country.

**Table 2:** Potential reduction of all-cause mortality among the low educated if they would have the risk factor prevalence of the high educated (Population Attributable Fraction (in %, with 95% CI)), by risk factor, country and sex.

	Behavioural risk factors						Social and economic risk factors					
	Smoking		Overweight		Physical inactivity		Lack of social participation		Low income		Economic inactivity	
Population	Men	Women	Men	Women	Men	Women	Men	Women	Men	Women	Men	Women
Finland	**8.0 (6.7–9.1)**	**3.5 (3.0–4.8)**	1.1 (0.9–1.6)	**3.4 (2.4–4.4)**	0.9 (0.7–1.2)	0.3 (0.0–0.8)	2.5 (2.4–2.6)	**1.9 (1.8–2.0)**	**9.6 (8.8–10.3)**	**4.4 (3.9–4.9)**	**3.3 (3.2–3.4)**	**3.2 (2.9–3.3)**
Sweden	**8.1 (6.3–9.8)**	**4.8 (3.5–6.2)**	**2.7 (1.7–3.6)**	**3.9 (2.9–4.7)**	na	na	1.9 (1.2–3.0)	**4.3 (3.2–5.2)**	**8.9 (8.0–9.6)**	**3.7 (3.2–4.2)**	2.8 (2.7–2.9)*	**3.0 (2.8–3.2)** *
Norway	***14.0 (11.7–16.1*** **)**	**8.8 (7.0–10.5)**	**3.5 (2.1–5.0)**	**4.2 (3.1–5.4)**	2.3 (1.4–3.3)	2.3 (1.7–2.9)	**3.1 (2.0–4.4)**	**4.6 (3.3–5.9)**	**9.3 (8.5–10.0)**	**5.7 (5.2–6.1)**	**3.5 (3.3–3.7)** *	**3.8 (3.5–4.1)** *
Denmark	**8.4 (6.8–9.9)**	**3.5 (2.8–4.4)**	**3.8 (2.8–4.7)**	**4.1 (3.6–4.8)**	2.4 (1.7–3.1)	**4.4 (1.5–3.3)**	**4.7 (3.1–6.1)**	**3.5 (2.0–5.2)**	**7.8 (7.3–8.1)**	**4.0 (3.7–4.2)**	na	na
England &W	***10.7 (9.6–11.9)***	**5.9 (5.0–6.9)**	**2.3 (1.5–3.1)**	**4.8 (4.0–5.4)**	na	na	**4.0 (2.9–5.1)**	**4.2 (3.0–5.2)**	**8.4 (7.9–8.8)**	**5.3 (4.9–5.5)**	1.7 (1.5–1.9)	2.0 (1.7–2.4)
Scotland	***11.1 (9.4–12.7)***	**9.9 (8.5–11.1)**	**2.5 (1.4–3.5)**	2.0 (1.2–3.0)	na	na	na	na	***10.0 (9.4–10.5)***	**5.2 (4.8–5.6)**	na	na
Netherlands	**5.5 (4.1–6.9)**	2.3 (1.8–2.9)	**3.7 (2.9–4.6)**	**4.3 (3.7–4.9)**	1.0 (0.3–1.8)	**3.2 (2.3–3.9)**	2.2 (1.2–3.4)	**3.1 (2.0–4.1)**	**8.6 (7.7–9.5)**	**4.8 (4.2–5.3)**	na	na
Belgium	**3.3 (2.5–4.3)**	0.8 (0.6–1.1)	**3.2 (2.5–3.9)**	**5.5 (4.9–6.0)**	2.6 (2.1–3.1)	**7.1 (6.3–7.8)**	**3.8 (2.4–5.1)**	1.6 (0.7–2.7)	**8.5 (8.1–8.8)**	**4.9 (4.7–5.1)**	na	na
France	**3.0 (2.4–4.3)**	0.9 (0.6–1.2)	**4.6 (3.9–5.4)**	**6.2 (5.5–6.9)**	**na**	na	**4.5 (3.2–5–5)**	**6.1 (5.1–7.1)**	**9.6 (8.6–10.5)**	**6.4 (5.8–6.9)**	na	na
Switzerland	**5.0 (3.1–7.1)**	0.7 (0.5–1.3)	**4.4 (3.4–5.4)**	**6.4 (5.8–7.0)**	**4.0 (3.0–5.0)**	**5.8 (4.8–6.8)**	na	na	**9.2 (7.6–10.8)**	**4.9 (4.2–5.7)**	**4.1 (3.9–4.3)** *	**3.1 (3.0–3.3)** *
Austria	**4.8 (3.2–7.5)**	0.6 (0.4–1.0)	**5.4 (3.7–6.9)**	**6.7 (5.8–7.7)**	**na**	na	2.6 (1.7–3.7)	**7.0 (5.9–7.9)**	na	na	1.6 (1.4–1.7)	2.4 (2.2–2.7)
Barcelona	2.6 (2.3–3.3)	0.5 (0.4–0.6)	2.7 (2.2–3.1)	**6.4 (5.9–6.9)**	2.6 (2.5–2.8)	2.8 (2.7–3.0)	**5.0 (4.1–5.8)**	2.1 (1.7–2.6)	na	na	na	na
Basque C	2.7 (1.9–4.1)	0.7 (0.4–1.0)	1.4 (0.7–2.1)	**5.8 (5.1–6.4)**	2.6 (2.5–2.7)	2.8 (2.7–3.0)	**5.0 (4.0–5.8)**	2.2 (1.7–2.6)	na	na	1.1 (1.0–1.1)	**5.6 (5.2–6.0)**
Madrid	2.4 (2.1–3.0)	0.4 (0.3–0.5)	2.6 (2.2–3.1)	**6.3 (5.9–6.8)**	2.6 (2.4–2.7)	2.7 (2.5.2.9)	**5.0 (4.1–5.9)**	2.0 (1.5–2.4)	na	na	**3.2 (3.1–3.3)** *	**4.7 (4.4–5.0)** *
Turin	1.7 (1.5–2.1)	0.2 (0.2–0.3)	**3.6 (3.4–3.8)**	**5.3 (5.1–5.4)**	1.2 (1.1–1.4)	2.0 (1.8–2.1)	**3.2 (2.1–4.4)**	1.4 (1.0–1.9)	na	na	**5.4 (5.2–5.5)** *	**3.7 (3.2–4.1)** *
Tuscany	1.6 (1.4–2.0)	0.3 (0.2–0.4)	**3.6 (3.4–3.8)**	**5.3 (5.1–5.4)**	1.2 (1.1–1.4)	2.0 (1.8–2.1)	**3.2 (2.0–4.5)**	1.4 (1.0–1.8)	na	na	na	na
Hungary	na	na	1.4 (0.5–2.5)	**4.5 (3.7–5.2)**	na	na	**5.1 (4.5–5.7)**	2.2 (1.7–2.8)	***10.6 (10.0–11.0)***	**6.1 (5.7–6.3)**	**4.9 (4.9–5.0)**	**3.5 (3.4–3.6)**
Czech R	***12.1 (9.2–14.9)***	2.7 (2.0–3.7)	**4.1 (2.4–5.7)**	**5.7 (4.3–7.0)**	0.0 (0.0–0.6)	1.6 (1.1–2.4)	na	na	**7.7 (6.2–9.1)**	**4.1 (3.5–4.7)**	**na**	**na**
Poland	***13.4 (12.2–14.3)***	2.6 (2.1–3.1)	1.0 (0.6–1.6)	**5.9 (5.3–6.4)**	na	na	**5.2 (4.9–5.5)**	2.1 (1.9–2.6)	***12.7 (11.6–13.6)***	**5.1 (4.4–5.7)**	**na**	**na**
Lithuania	**8.2 (6.7–9.5)**	2.1 (1.4–3.0)	1.3 (0.4–2.3)	2.0 (1.6–2.4)	**3.4 (2.6–4.2)**	**6.8 (6.0–7.9)**	na	na	na	na	na	na
Estonia	**8.4 (6.7–10.4)**	**3.1 (2.0–4.5)**	0.1 (0.0–0.1)	**5.6 (4.0–7.1)**	1.5 (1.1–2.8)	2.8 (1.5–4.3)	na	na	na	na	na	na
All #	**6.8**	**2.2**	2.7	**5.3**	2.4	2.8	**3.9**	**2.2**	**9.2**	**4.9**	3.3	**3.4**

* economically inactive include unemployed.

na: data not available.

# Median value for all populations included in the analysis.

Normal font style: PAF less than 3%.

**Bold font style**: PAF between 3 and 10%.

***Bold italic font style***: PAF at least 10%.

As indicated by their non-overlapping 95% CIs, the observed differences in PAFs between countries are often unlikely to be due to random error. For example, reducing smoking prevalence to the level of the high educated will prevent a considerably larger fraction of deaths among the low educated in the North, West and Centre/East than in the South. This is due to the fact that inequalities in smoking prevalence are larger in the former than in the latter regions (see supporting information: Table S6 in File S1).

**Figure 1:**
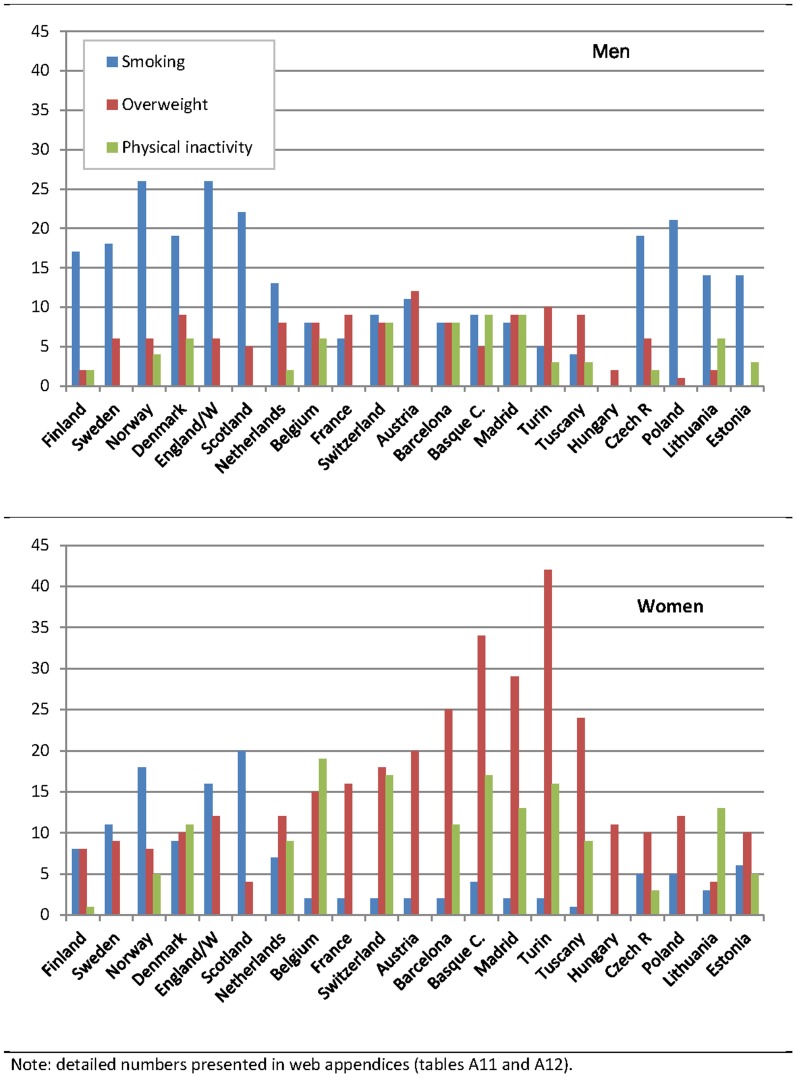
Potential reduction of relative educational inequalities in all-cause mortality between low and high educated (in %), upward levelling scenario according to smoking, overweight and physical activity by country and sex.

**Figure 2:**
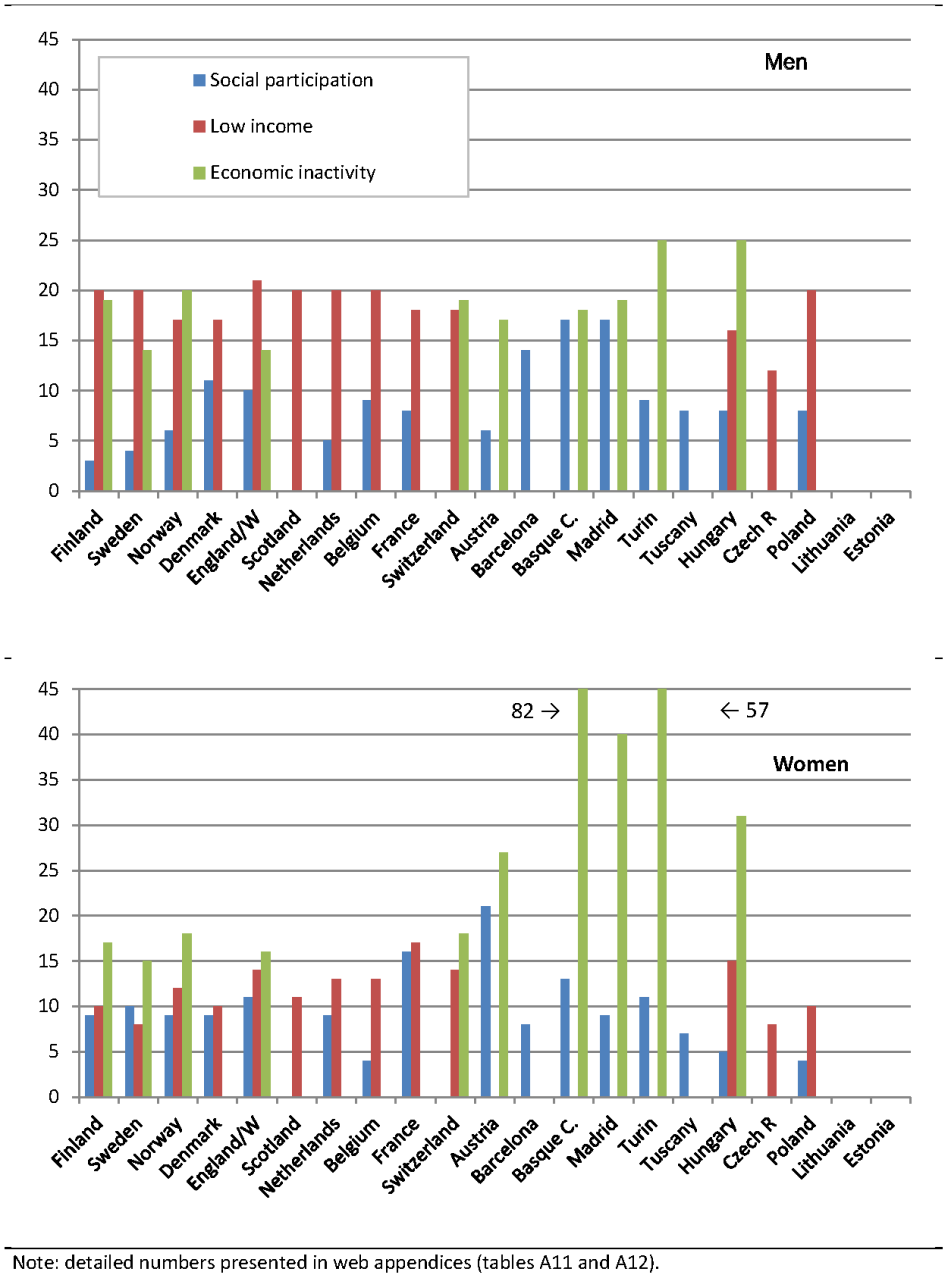
Potential reduction of relative educational inequalities in all-cause mortality between low and high educated (in %), upward levelling scenario according to social participation, low income and economic inactivity by country and sex.

We present the impact of upward levelling and best practice scenarios on *absolute* inequalities in mortality in tables 3 and 4, respectively. Both tables visualize the potential impact across risk factors by use of colours ranging from yellow (none or minor impact) through light green to dark green (major impact). Reductions in *relative* inequalities for the same scenarios are presented in Figure 1 and Figure 2.

**Table 3:** Potential reduction of absolute educational inequalities in all-cause mortality between low and high educated (in deaths per 100,000 person-years), upward levelling scenario, by risk factor, country and sex.

	Behavioural risk factors						Social and economic risk factors					
	Smoking		Over-weight		Physical inactivity		Social participation		Low income		Economic inactivity	
Population	M	W	M	W	M	W	M	W	M	W	M	W
Finland	***102***	**21**	15	**20**	11	2	16	**23**	***121***	**26**	**114**	**44**
Sweden	**76**	**28**	**25**	**22**	na	na	17	**25**	***83***	**22**	**59** *	**39** *
Norway	***152***	**55**	**37**	**25**	**25**	15	**33**	**29**	***101***	**35**	***113*** *	**56** *
Denmark	***105***	**30**	**49**	**32**	**30**	**34**	**60**	**28**	***96***	**32**	na	na
England/W	***108***	**38**	**23**	**30**	na	na	**41**	**27**	***84***	**33**	**58**	**39**
Scotland	***125***	**74**	**28**	15	na	na	na	na	***111***	**39**	na	na
Netherlands	**54**	12	**36**	**23**	10	17	**20**	16	***84***	**25**	na	na
Belgium	**36**	4	**34**	**29**	**29**	**38**	**41**	9	***91***	**26**	na	na
France	**33**	4	**51**	**27**	na	na	**49**	**27**	***105***	**28**	na	na
Switzerland	**49**	4	**45**	**29**	**40**	**27**	na	na	***92***	**23**	***98*** *	**28** *
Austria	**59**	4	**66**	**38**	na	na	**31**	**40**	na	na	***96***	**51**
Barcelona	**24**	2	**26**	**24**	**25**	11	**46**	8	na	na	na	na
Basque C.	**25**	3	13	**21**	**25**	10	**46**	8	na	na	**47**	**50**
Madrid	**26**	2	**28**	**27**	**28**	11	**52**	8	na	na	**58** *	**36** *
Turin	14	1	**30**	**21**	10	8	**27**	6	na	na	**75** *	**29** *
Tuscany	13	1	**28**	**21**	10	8	**26**	6	na	na	na	na
Hungary	na	na	**37**	**47**	na	na	***137***	**23**	***284***	**63**	***453***	133
Czech R	***216***	**22**	**69**	**45**	**26**	14	na	na	***137***	**33**	na	na
Poland	***273***	**22**	19	**48**	na	na	***106***	17	***258***	**41**	na	na
Lithuania	***200***	16	**30**	**21**	***83***	**69**	na	na	na	na	na	na
Estonia	***237***	**38**	4	**67**	**41**	**35**	na	na	na	na	na	na
All ^#^	**68**	14	**30**	**27**	**26**	14	**41**	**20**	***101***	**32**	**96**	**50**

Notes: na: data not available.

# Median value of all populations included in the analysis.

*Economically inactive include unemployed.

M = men, W = women.

Normal font style: reduction of mortality rate difference between low and high educated by 0–19 deaths per 100,000.

**Bold font style**: reduction of mortality rate difference between low and high educated by 20–79 deaths per 100,000.

***Bold italic font style***: reduction of mortality rate difference between low and high educated by at least 80 deaths per 100,000.

**Table 4:** Potential reduction of absolute educational inequalities in all-cause mortality between low and high educated (in deaths per 100,000 person-years), best practice scenario, by risk factor, country and sex.

	Behavioural risk factors						Social and economic risk factors					
	Smoking		Over-weight		Physical inactivity		Social participation		Low income		Economic inactivity	
Population	M	W	M	W	M	W	M	W	M	W	M	W
Finland	**55**	18	ref	0	ref	ref	−12	5	**20**	1	12	ref
Sweden	**33**	**58**	0	0	na	na	0	1	14	6	ref*	ref*
Norway	***83***	***80***	1	0	**72**	**73**	14	1	**48**	**26**	13*	13*
Denmark	***204***	***101***	5	0	15	**23**	16	1	**70**	**39**	na	na
England/W	**68**	**79**	**34**	17	na	na	7	1	**21**	12	**ref**	**40**
Scotland	***92***	***120***	**24**	ref	na	na	na	na	**27**	10	na	na
Netherlands	**76**	**52**	0	0	**41**	**39**	**ref**	0	14	2	na	na
Belgium	**97**	**32**	0	0	**31**	**40**	4	**ref**	**27**	11	na	na
France	**ref**	4	3	0	na	na	17	7	13	6	na	na
Switzerland	***87***	**25**	0	0	15	17	na	na	19	10	0*	18*
Austria	3	5	10	0	na	na	16	13	na	na	17	**54**
Barcelona	18	−2	9	3	**59**	**32**	**32**	4	na	na	na	na
Basque C.	11	3	2	0	**52**	**23**	**31**	3	na	na	5	**45**
Madrid	14	−4	10	3	**62**	**33**	**36**	4	na	na	1*	**25** *
Turin	11	ref	0	0	**40**	18	19	0	na	na	**34** *	**21** *
Tuscany	12		0	0	**39**	**26**	18	2	na	na	na	na
Hungary	na	na	**35**	7	na	na	***138***	**21**	**25**	13	***293***	***130***
Czech R	***114***	**31**	**35**	16	***140***	***105***	na	na	ref	ref	na	na
Poland	***226***	**39**	**29**	14	na	na	***153***	**48**	14	8	na	na
Lithuania	***193***	1	−14	−6	***121***	***112***	na	na	na	na	na	na
Estonia	***256***	**42**	−8	**37**	***159***	***124***	na	na	na	na	na	na
All #	***76***	**32**	2	0	**52**	**33**	17	3	**20**	10	14	**50**

Notes: na: data not available.

<?ENTCHAR num?> Median value of all populations included in the analysis.

*Economically inactive include unemployed (England/Wales and Finland is reference country for men and women respectively). Sweden is reference of countries with unemployed included in the active, both among men and women.

M = men, W = women.

Normal font style: reduction of mortality rate difference between low and high educated by 0–19 deaths per 100,000.

**Bold font style**: reduction of mortality rate difference between low and high educated by 20–79 deaths per 100,000.

***Bold italic font style***: reduction of mortality rate difference between low and high educated by at least 80 deaths per 100,000.

### Upward levelling scenarios

A complete elimination of inequalities in risk factors, by *upward levelling of the prevalence of risk factors* to the level currently seen in the highest education group, often results in a substantial reduction of absolute inequalities in mortality (table 3). However, this depends on the chosen risk factor and varies substantially between countries.

In most countries one can expect a notable decrease of inequalities in mortality among men, often by more than 80 deaths per 100 000 person years, if differences in smoking between educational groups would disappear, particularly in the North and Centre/East. However, the potential reduction is much smaller among women: outside the Nordic countries, the reduction of inequalities is often less than 20 deaths per 100 000 person years. Among men, elimination of inequalities in overweight or physical inactivity often has a smaller effect than elimination of inequalities in smoking, but among women overweight is often more important than smoking.

Among the social and economic risk factors, low income and economic inactivity are more important for tackling inequalities in mortality than lack of social participation, and also are often more important than smoking. Elimination of inequalities in low income would reduce inequalities in mortality by more than 80 deaths per 100 000 person years among men in all countries with available data. Upward levelling of the proportion of economically inactive people, in countries for which these data are available, also substantially reduces inequalities in mortality, particularly among Hungarian men and women (by 453 and 133 deaths per 100 000 person years, respectively).

The results for relative inequalities in mortality sometimes lead to a rather different picture of variation between countries (Figure 1 and Figure 2). The main reason is that relative inequalities in mortality do not vary between countries in the same pattern as absolute inequalities in mortality. For example, due to high average mortality rates absolute inequalities in mortality are particularly large in the Centre/East. As a result the reduction of the mortality Rate Difference is sometimes (e.g., in the case of smoking) largest here (table 3) but the reduction of the mortality Rate Ratio (Figure 1) is not. On the other hand, due to low average mortality rates the reduction of absolute inequalities is often small in the South (table 3) even if the reduction of relative inequalities is large (e.g., in the case of overweight (Figure 1) and economic inactivity (Figure 2)).

### Best practice scenarios

Except from being a more realistic scenario, the best practice scenario differs from the upward levelling scenario in that the “best practice group” no longer stems from the same country (i.e., the highest educated group in each country), but from another country, and that the risk factor exposure among all educational levels is changed, not only that among the lower educated. This explains why best practice scenarios sometimes have larger effects than upward levelling scenarios. It should also be noted that the best practice scenarios sometimes have "negative" effects, in the sense that inequalities in mortality go up instead of down. This is the case when countries have a high average risk factor prevalence, and therefore could not be selected as best practice country, but at the same time have smaller inequalities in risk factor prevalence than the best practice country.

When we compare tables 3 and 4, the first thing to note is that best practice scenarios have a much smaller impact on inequalities in mortality than upward levelling scenarios for overweight, social participation, low income and economic inactivity (women only), but not for smoking and physical inactivity. These best practice scenarios suggest that substantial reductions (more than 20 or even 80 deaths per 100 000 person years) of absolute inequalities in mortality can realistically be achieved in many countries through smoking and physical inactivity, both among men and women (table 4). The distribution of income across educational groups is very similar across European countries. Therefore, reducing income inequalities to the level of the best practice country (the Czech Republic) will have only a minor impact on inequalities in mortality.

Still, small reductions of absolute inequalities may go together with sizable reductions in relative inequalities when average mortality levels are high, as in the case of Central/Eastern Europe (see supporting information: Table S9 in File S1).

## Discussion

Our study has a number of important strengths, based on its wide geographical coverage and the application of straightforward quantitative methods. It explores a novel approach to identify entry-points for policies to tackle health inequalities, but in the implementation of this approach we encountered several limitations that all indicate a need for further research.

First, the scope of this study was limited by data availability. We could only include one indicator of socioeconomic position, and due to restrictions with regard to available data and/or available scientific knowledge we were unable to include the full range of potentially relevant risk factors in our analysis. Not all behavioural risk factors could be included, alcohol consumption being the prime example of a risk factor known to contribute importantly to inequalities in mortality in many European countries [3], [44], [45] but for which no reliable survey data are available. Specific material living conditions, such as those related to housing, work or environmental pollution, could not be included either. With the exception of social participation, psychosocial risk factors such as those relating to psychosocial stress [46], [47] were largely absent from the analysis as well.

Also, we only studied risk factors one-by-one, because available methods for combining them assume mutual independence [48] which would not be guaranteed in our case, e.g. because ‘downstream’ risk factors, such as smoking, are nested within the ‘upstream’ ones, such as low income. In an additional analysis reported elsewhere, we combined smoking and obesity, and showed that the combined effect of eliminating inequalities in both risk factors considerably exceeded the effects of each apart [42], [43]. This implies that the full potential for reducing inequalities in mortality may well be even larger than the separate estimates for the six single risk factors in our analysis suggest.

Second, while we have undertaken major harmonization efforts some potential comparability problems remain, e.g. related to the fact that mortality data for some countries are based on unlinked cross-sectional studies [49] and that data from a few countries could only be obtained from regions. However, educational differences in mortality in Turin and Tuscany are of the same magnitude as differences in Italy altogether [50], and the same applies to the Spanish regions and the whole of Spain [51]. We also compared the overall mortality rates in corresponding ages and corresponding years for men and women obtained from our regional data to national data available in the Human Mortality Database (HMD) (http://www.mortality.org/). The rates corresponded well to the national averages. We thus believe that our results on educational differences in disability-free life expectancy are not crucially affected by the use of regional mortality data instead of national data. Furthermore, the comparability of our economic inactivity data was clearly suboptimal because temporary unemployed were classified with the inactive in Norway, Sweden, Switzerland, and Madrid, and with the active in all other populations. This implies that the causal effect of economic activity on mortality will be different between these two groups of countries. However, different RRs were applied for these groups and we could also not find any systematic differences in reduction of mortality inequalities between countries with different classifications of the unemployed (table 3). Furthermore, the reasons for economic inactivity may vary between countries. The most common reason for economic inactivity among men is long term illness or disability, which could give rise to reverse causation [52]–[54], but they could also be studying for a qualification, staying at home to look after their family, or be retired [55]. The latter is less likely to be the case in our data given the restricted age range in our analyses. Among women, the main reason is that they look after their family, which is particularly common in the South. Despite these potential comparability problems, the data that we have used represent the best available data, and our main results are consistent with patterns that have been reported before on the basis of cause-specific mortality analyses [3].

A third group of limitations relates to the assumptions and uncertainties of the counterfactual estimations. It is of course debatable whether just by reducing the level of one risk factor to the level of the higher educated, this would prevent the number of deaths among the lower educated that we report here. Some of the assumptions underlying the method may be controversial [43]. We assume causality from risk factors to mortality (which is relatively unproblematic because we relied on systematic reviews that have tried to filter out the causal relationship between risk factors and mortality) but also from education to risk factors which is more uncertain. We also assume that the relative risks for the risk factors are the same for all countries, as there are no high-quality literature reviews on the impact of risk factors for each country available. Fortunately, an increasing body of evidence suggests that, when the metric of exposure is comparable, the relative risks are similar across populations in different world regions [56]. We further assume that the relative risks of the risk factors are the same for all educational groups. With respect to smoking, the Whitehall II study has suggested that smoking may be more harmful for those placed lower in the social hierarchy [57], but there is no systematic data on how the impact of proximate risk factors differs by socioeconomic group [43].

Furthermore, the exposure data have not always been collected for a point in time that allowed a lag-time before mortality effects occur. Take smoking as an example. Whether or not we have systematically under- or overestimated the potential for reducing inequalities in mortality by taking too recent prevalence data depends on whether changes in prevalence of smoking by education have occurred during this lag-time which, for some of the effects of smoking, may be 20 years or more. We know that in countries which are far advanced in the smoking epidemic inequalities in smoking behaviour have been increasing over time, especially among women [58]. However, in a study reported elsewhere we replaced current by historical smoking prevalence rates from the early 1980s for England and France. We found that in the upward levelling scenario the potential reduction of relative inequalities in mortality remained almost the same for English men and French men and women, while it declined from 16% to 8% among English women, suggesting that among the latter we may have overestimated the potential for reducing inequalities in mortality [59]. However, as this is an extreme case (not all risk factors have such long lag-times, and not all risk factors have equally dynamic distributional changes over time), most of our results are unlikely to be seriously affected by this problem.

Our analysis shows that in Europe smoking is the single most important ‘downstream’ risk factor for educational inequalities in mortality among men, and overweight is the most important among women. For smoking, this is in line with individual-level studies which have generally found important contributions of smoking to inequalities in mortality [4], [6]–[8], but it is important to note that most high-quality studies come from a small number of countries, and that our study therefore considerably expands the knowledge-base. We found substantial regional variations in the importance of smoking, in line with previous findings from the 1990s [3]: educational inequalities in smoking were and still are more important in the Centre/East (particularly among men) and in the North, and less important (or even “reverse”) in the South, probably because of differences in the progression of the smoking epidemic [17], [58], [60].

The relative importance of other ‘downstream’ risk factors than smoking has been less frequently studied, but previous studies have shown that overweight and obesity and physical inactivity contribute to the explanation of inequalities in mortality [4], [6]–[8]. We have taken this one step further by expanding geographic coverage and introducing a comparative perspective. Due to the fact that overweight and physical inactivity are more strongly socially patterned among women in the South than among women elsewhere in Europe [23], [61], perhaps because of traditional gender roles [61], these two risk factors are relatively important entry-points for policy in that part of the subcontinent.

Our findings for social and economic risk factors illustrate that distal or ‘upstream’ risk factors, particularly low income and economic inactivity, are also important entry-points for policies to tackle health inequalities. The role of upstream risk factors, such as income inequalities, economic activity status, and other aspects of socio-economic inequalities in generating health inequalities has been well documented [2], [35], [62], [63]. Welfare states provide a variety of social transfers (such as housing related benefits, unemployment, pensions, and sickness and disability benefits) as well as key services (most notably health care or social services), which may help to modify the impact of upstream risk factors on health [36], [64], [65]. The principles underpinning welfare states and the generosity of social transfers and entitlements vary extensively between welfare state regimes [65], and these variations may partly account for differences in the patterning of health inequalities [66], [67]. A causal effect of these upstream factors on mortality is less well established, however, so caution must be exercised in interpreting our findings. In the absence of experimental evidence on the impact of levelling income on inequalities in health, it is important to note that while inequalities in mortality are not smaller in countries with smaller income inequalities [3], [68], they are smaller in countries with smaller inequalities in smoking [3], [69], [70]. Further study is therefore necessary to corroborate our findings.

The upward-levelling scenarios demonstrated that there is a great theoretical potential for reducing inequalities in mortality in most European countries, for example by tobacco control policies, income redistribution, housing policies, labour market policies and social policies particularly aimed at those with less resources [66], [67]. The best practice scenarios often produced considerably smaller reductions in mortality inequalities than the upward levelling scenarios, but these were still substantial in many countries for smoking and physical inactivity. This suggests that achieving the theoretical potential may be feasible in some areas, but that in other areas more investments are needed in the development of effective interventions and policies to reduce inequalities in health. The results of the best-practice scenarios should also be interpreted in light of the fact that European countries are characterized by a large diversity of political histories [71], which may hinder policy learning and policy transfers between countries.

In any event, our study shows that each country needs its own tailored strategy for tackling health inequalities, and our study methods can be used to identify each country's main priorities for action. We did not find one country that had the smallest inequalities in all determinants of health inequalities. Instead, most countries can serve as an example for others, and inequalities in mortality could probably be reduced substantially if countries were willing and able to more systematically exchange experiences with tackling health inequalities.

## Supporting Information

File S1 **File includes Tables S1–S12.** Table S1: Characteristics of the mortality data. Table S2: Rate ratios (from the EURO-GBD-SE mortality data set) for the association between education and all-cause mortality. Table S3: Relative risks for the impact of risk factors on all-cause mortality. Table S4: Sources of prevalences. Table S5: Prevalences of smoking. Table S6: Prevalences of overweight. Table S7: Prevalences of physical inactivity. Table S8: Prevalences of social participation. Table S9: Prevalences of lower income quartiles. Table S10: Prevalences of economic inactivity. Table S11: Potential reduction (in %) of relative educational inequalities in all-cause mortality between low and high educated, upward levelling scenario, by risk factor, country and sex. Table S12: Potential reduction (in %) of relative educational inequalities in all-cause mortality between low and high educated, best practice scenario, by risk factor, country and sex.
(DOCX)
