# Supplementary material for: How Can Inequalities in Mortality Be Reduced? A Quantitative Analysis of 6 Risk Factors in 21 European Populations
Source: PLoS One. 2014 Nov 4;9(11):e110952. doi: 10.1371/journal.pone.0110952 (PMC4219687; doi:10.1371/journal.pone.0110952)
Supplement: File S1 — File includes Tables S1–S12. Table S1: Characteristics of the mortality data. Table S2: Rate ratios (from the EURO-GBD-SE mortality data set) for the association between education and all-cause mortality. Table S3: Relative risks for the impact of risk factors on all-cause mortality. Table S4: Sources of prevalences. Table S5: Prevalences of smoking. Table S6: Prevalences of overweight. Table S7: Prevalences of physical inactivity. Table S8: Prevalences of social participation. Table S9: Prevalences of lower income quartiles. Table S10: Prevalences of economic inactivity. Table S11: Potential reduction (in %) of relative educational inequalities in all-cause mortality between low and high educated, upward levelling scenario, by risk factor, country and sex. Table S12: Potential reduction (in %) of relative educational inequalities in all-cause mortality between low and high educated, best practice scenario, by risk factor, country and sex. (DOCX) [file pone.0110952.s001.docx]

**SUPPORTING INFORMATION**

Table S1: Characteristics of the mortality data

| Population | Type of dataset | Period | Geographic  coverage | Demographic  coverage |
| --- | --- | --- | --- | --- |
| Finland | longitudinal | 2001–2007 | national | 20% of Finns are excluded (at random) |
| Sweden | longitudinal | 2001–2006 | national | whole population |
| Norway | longitudinal | 2001–2006 | national | whole population |
| Denmark | longitudinal | 2001–2005 | national | whole population |
| England & Wales | longitudinal | 2001–2006 | national | 1% of the population |
| Scotland | longitudinal | 2001–2006 | national | 5.3% representative sample of the population |
| Netherlands | longitudinal | 1998–2003,  1999–2004,  2000–2005,  2001–2006,  2002–2007 | national | linkage based on the labour force survey |
| Belgium | longitudinal | 2001–2005 | national | whole population |
| France | longitudinal | 1999–2005 | national | 1% of the population, born outside France mainland excluded |
| Switzerland | longitudinal | 2001–2005 | national | Non-Swiss nationals excluded |
| Austria | longitudinal | 2001–2002 | national | whole population |
| Barcelona | cross-sectional linked | 2000–2006 | city | whole population |
| Basque Country | longitudinal | 2001–2006 | region | whole population |
| Madrid | cross-sectional linked | 2001–2003 | region | whole population |
| Turin | longitudinal | 2001–2006 | city | whole population |
| Tuscany | longitudinal | 2001–2005 | Florence,  Leghorn,  Prato | whole population |
| Hungary | cross-sectional unlinked | 1999–2002 | national | whole population |
| Czech Republic | cross-sectional unlinked | 1999–2003 | national | whole population |
| Poland | cross-sectional unlinked | 2001–2003 | national | whole population |
| Lithuania | longitudinal | 2001–2005 | national | whole population |
| Estonia | cross-sectional unlinked | 1998–2002 | national | whole population |

**Table S2: Rate ratios (from the EURO-GBD-SE mortality data set) for the association between education and all-cause mortality**

|  | | **MEN** | | | | **WOMEN** | | | |
| --- | --- | --- | --- | --- | --- | --- | --- | --- | --- |
|  | | age | age | age | age | age | age | age | age |
|  |  | 30-44 | 45-59 | 60-69 | 70-79 | 30-44 | 45-59 | 60-69 | 70-79 |
| Finland | Low | 4.27 | 2.35 | 1.82 | 1.55 | 3.49 | 2.05 | 1.59 | 1.50 |
|  | Mid | 2.27 | 1.76 | 1.50 | 1.26 | 1.69 | 1.32 | 1.25 | 1.22 |
| Sweden | Low | 3.40 | 2.25 | 1.75 | 1.54 | 2.68 | 2.08 | 1.82 | 1.56 |
|  | Mid | 1.90 | 1.68 | 1.39 | 1.26 | 1.49 | 1.45 | 1.42 | 1.27 |
| Norway | Low | 3.95 | 2.79 | 2.15 | 1.72 | 3.01 | 2.40 | 1.97 | 1.65 |
|  | Mid | 2.05 | 1.78 | 1.52 | 1.32 | 1.77 | 1.44 | 1.39 | 1.22 |
| Denmark | Low | 3.93 | 2.37 | 1.69 | 1.45 | 2.73 | 1.98 | 1.74 | 1.41 |
|  | Mid | 1.99 | 1.63 | 1.41 | 1.32 | 1.35 | 1.36 | 1.31 | 1.19 |
| England &W | Low | 1.95 | 2.12 | 1.59 | 1.56 | 1.80 | 1.37 | 1.77 | 1.63 |
|  | Mid | 1.62 | 1.54 | 1.16 | 1.13 | 0.98 | 0.88 | 1.24 | 1.16 |
| Scotland | Low | 4.63 | 2.55 | 1.78 | 1.78 | 3.41 | 1.94 | 1.95 | 1.80 |
|  | Mid | 2.71 | 1.54 | 1.40 | 1.35 | 1.18 | 1.13 | 1.20 | 1.23 |
| Netherlands | Low | 2.03 | 1.93 | 1.60 | 1.83 | 1.87 | 1.67 | 1.72 | 1.34 |
|  | Mid | 1.32 | 1.24 | 1.28 | 1.43 | 1.05 | 1.24 | 1.28 | 0.97 |
| Belgium | Low | 2.62 | 1.92 | 1.75 | 1.55 | 2.00 | 1.56 | 1.56 | 1.55 |
|  | Mid | 1.73 | 1.40 | 1.35 | 1.22 | 1.37 | 1.27 | 1.30 | 1.20 |
| France | Low | 3.73 | 2.49 | 1.91 | 2.04 | 3.17 | 1.90 | 1.46 | 1.35 |
|  | Mid | 2.36 | 1.86 | 1.39 | 1.56 | 1.90 | 1.45 | 1.16 | 1.14 |
| Switzerland | Low | 3.77 | 2.64 | 2.12 | 1.69 | 2.52 | 1.63 | 1.36 | 1.48 |
|  | Mid | 1.91 | 1.64 | 1.47 | 1.27 | 1.24 | 1.12 | 1.06 | 1.17 |
| Austria | Low | 2.90 | 2.39 | 1.86 | 1.53 | 2.27 | 1.65 | 1.32 | 1.48 |
|  | Mid | 1.79 | 1.88 | 1.51 | 1.27 | 1.43 | 1.29 | 1.10 | 1.18 |
| Barcelona | Low | 4.24 | 1.81 | 1.40 | 1.29 | 2.80 | 1.22 | 1.22 | 1.32 |
|  | Mid | 1.89 | 1.30 | 1.10 | 1.08 | 1.35 | 1.01 | 1.09 | 1.07 |
| Basque C | Low | 3.19 | 1.66 | 1.27 | 1.19 | 2.40 | 1.28 | 0.96 | 1.18 |
|  | Mid | 1.62 | 1.20 | 1.15 | 1.03 | 1.61 | 1.16 | 0.91 | 1.04 |
| Madrid | Low | 3.09 | 1.76 | 1.31 | 1.20 | 1.89 | 1.04 | 1.33 | 1.30 |
|  | Mid | 1.63 | 1.39 | 1.24 | 1.10 | 1.60 | 1.02 | 1.37 | 1.20 |
| Turin | Low | 2.93 | 1.64 | 1.81 | 1.31 | 2.06 | 1.34 | 1.05 | 1.04 |
|  | Mid | 1.38 | 1.17 | 1.23 | 1.08 | 1.36 | 1.26 | 0.98 | 1.01 |
| Tuscany | Low | 4.79 | 2.18 | 1.59 | 1.36 | 2.13 | 1.34 | 1.32 | 1.14 |
|  | Mid | 2.07 | 1.45 | 1.25 | 1.10 | 1.60 | 1.16 | 1.07 | 0.99 |
| Hungary | Low | 6.83 | 4.81 | 2.50 | 1.92 | 4.25 | 2.45 | 1.55 | 1.26 |
|  | Mid | 3.00 | 1.79 | 1.40 | 1.12 | 2.04 | 1.46 | 0.98 | 0.81 |
| Czech R | Low | 3.75 | 3.62 | 2.95 | 2.40 | 2.71 | 2.49 | 2.22 | 2.01 |
|  | Mid | 1.69 | 1.81 | 1.57 | 1.36 | 1.47 | 1.55 | 1.65 | 1.60 |
| Poland | Low | 7.95 | 3.68 | 2.42 | 2.01 | 5.00 | 2.22 | 2.00 | 1.69 |
|  | Mid | 3.04 | 2.43 | 2.13 | 1.80 | 2.03 | 1.84 | 1.73 | 1.42 |
| Lithuania | Low | 3.97 | 3.12 | 2.05 | 1.66 | 4.73 | 2.85 | 1.84 | 1.55 |
|  | Mid | 2.21 | 2.02 | 1.51 | 1.36 | 1.80 | 1.55 | 1.34 | 1.31 |
| Estonia | Low | 6.71 | 3.24 | 2.15 | 1.61 | 6.84 | 3.12 | 2.02 | 1.57 |
|  | Mid | 3.17 | 2.34 | 1.82 | 1.46 | 2.14 | 1.91 | 1.63 | 1.41 |

**Table S3: Relative risks for the impact of risk factors on all-cause mortality**

|  | **MEN** | | | | | **WOMEN** | | | |
| --- | --- | --- | --- | --- | --- | --- | --- | --- | --- |
|  | age 30-44 | | age 45-59 | age 60-69 | age 70-79 | age 30-44 | age 45-59 | age 60-69 | age 70-79 |
| **Smoking** |  | |  |  |  |  |  |  |  |
| Current | 2.07 | | 2.07 | 2.07 | 2.07 | 1.74 | 1.74 | 1.74 | 1.74 |
| Former | 1.35 | | 1.35 | 1.35 | 1.35 | 1.23 | 1.23 | 1.23 | 1.23 |
| Never | 1 | | 1 | 1 | 1 | 1 | 1 | 1 | 1 |
| **Overweight** |  | |  |  |  |  |  |  |  |
| 30+ | 1.55 | | 1.54 | 1.52 | 1.50 | 1.50 | 1.49 | 1.48 | 1.45 |
| 25-30 | 1.20 | | 1.20 | 1.19 | 1.18 | 1.15 | 1.15 | 1.14 | 1.14 |
| -25 | 1 | | 1 | 1 | 1 | 1 | 1 | 1 | 1 |
| **Physical inactivity** |  | |  |  |  |  |  |  |  |
| Sedentary | 1.28 | | 1.28 | 1.28 | 1.28 | 1.54 | 1.54 | 1.54 | 1.54 |
| Active | 1 | | 1 | 1 | 1 | 1 | 1 | 1 | 1 |
| **Social participation** |  | |  |  |  |  |  |  |  |
| No participation | 1.22 | | 1.22 | 1.22 | 1.22 | 1.22 | 1.22 | 1.22 | 1.22 |
| Participation | 1 | | 1 | 1 | 1 | 1 | 1 | 1 | 1 |
| **Low income** |  | |  |  |  |  |  |  |  |
| Lowest quart. | 1.29 | | 1.29 | 1.29 | 1.29 | 1.19 | 1.19 | 1.19 | 1.19 |
| Second quart. | 1.16 | | 1.16 | 1.16 | 1.16 | 1.08 | 1.08 | 1.08 | 1.08 |
| Third quart. | 1.08 | | 1.08 | 1.08 | 1.08 | 1.05 | 1.05 | 1.05 | 1.05 |
| Highest quart. | 1 | | 1 | 1 | 1 | 1 | 1 | 1 | 1 |
| **Economic inactivity** | | | | |  |  |  |  |  |
| Inactive (excluding unemployed) | | 4.37 | 3.36 | 1 | 1 | 1.94 | 2.10 | 1 | 1 |
| Inactive (including unemployed) | | 4.38 | 3.73 | 1 | 1 | 2.68 | 3.14 | 1 | 1 |
| Active | | 1 | 1 | 1 | 1 | 1 | 1 | 1 | 1 |

Sources: smoking ([40](#_ENREF_40)), overweight ([39](#_ENREF_39)), physical activity ([18](#_ENREF_18)), social participation ([29](#_ENREF_29)), income quartiles ([57](#_ENREF_57)) and economic inactivity (own mortality data).

**Table S4: Sources of prevalences^1^**

| Country | Name of surveys | Years | N |
| --- | --- | --- | --- |
|  |  |  |  |
| Austria | European Community Household Survey. wave 7 | 2000 | 5801 |
| Belgium | Health Interview Survey 1997 + 2001 | 97/01 | 18481 |
| Czech Republic | Sample Survey of the Health Status of the Czech Population | 2002 | 2476 |
| Denmark | Danish Health and Morbidity Survey 2000 | 2000 | 16690 |
| England & Wales | English Health Survey 2001 | 2001 | 15767 |
| Estonia | Health Behavior among Estonian Adult Population | 02/04 | 4376 |
| Finland | Finbalt Health Monitor | 94/98/00/02/04 | 20371 |
| France | National Health Survey (Enquête Décennale Santé) (Insee).^2^ | 2004 | 17828 |
| Italy | Health and health care utilization 1999-2000 | 99-00 | 118245 |
| Lithuania | Finbalt Health Monitor | 94/98/00/02/04 | 11647 |
| Netherlands | Permanent Onderzoek Leefsituatie (POLS) | 03/04 | 15803 |
| Norway | Statistics Norway/Norwegian Directorate of Health | 1998-2002 | 5738 |
| Poland | Second nationwide sample survey of the health status of the Polish population | 2004 | 35248 |
| Scotland | Scottish Health Survey | 2003 | 6912 |
| Spain | National Health Survey 2001 | 2001 | 20748 |
| Basque Country | Health Survey of the Basque Country | 2002 | 8920 |
| Sweden | Swedish Survey of Living Conditions | 00-01 | 11484 |
| Switzerland | Swiss Health Survey | 2002 | 19511 |

^1^ Prevalence data of social participation and income stem from the first (<http://www.europeansocialsurvey.org/data/download.html?r=1>) and (<http://www.europeansocialsurvey.org/data/download.html?r=2>) second round of the European Social Survey. Prevalence data of economic activity stem from the EURO-GBD-SE mortality data (<http://www.euro-gbd-se.eu/fileadmin/euro-gbd-se/public-files/EURO-GBD-SE_Final_report.pdf>).

^2^ Source: Santé - 2003 (standard version) - (2003) [electronic file]. INSEE [data producer]. Centre Maurice Halbwachs (CMH) [data distributer].

**Table S5: Prevalences of smoking**

|  |  | **Men** | | | |  | **Women** | | | |
| --- | --- | --- | --- | --- | --- | --- | --- | --- | --- | --- |
|  | **age** | 30-44 | 45-59 | 60-69 | 70-79 |  | 30-44 | 45-59 | 60-69 | 70-79 |
| **Education** | **Smoking** | **AUSTRIA** | |  |  |  |  |  |  |  |
| Low | Current | 0.591 | 0.354 | 0.182 | 0.096 |  | 0.338 | 0.198 | 0.065 | 0.017 |
|  | Former | 0.148 | 0.297 | 0.402 | 0.443 |  | 0.130 | 0.186 | 0.124 | 0.054 |
|  | Never | 0.261 | 0.349 | 0.416 | 0.460 |  | 0.532 | 0.616 | 0.811 | 0.928 |
| Middle | Current | 0.497 | 0.373 | 0.251 | 0.165 |  | 0.349 | 0.259 | 0.136 | 0.061 |
|  | Former | 0.179 | 0.281 | 0.369 | 0.429 |  | 0.183 | 0.156 | 0.164 | 0.193 |
|  | Never | 0.325 | 0.347 | 0.380 | 0.406 |  | 0.469 | 0.586 | 0.700 | 0.746 |
| High | Current | 0.198 | 0.331 | 0.189 | 0.053 |  | 0.164 | 0.267 | 0.063 | 0.005 |
|  | Former | 0.176 | 0.339 | 0.379 | 0.326 |  | 0.207 | 0.216 | 0.195 | 0.164 |
|  | Never | 0.626 | 0.330 | 0.432 | 0.621 |  | 0.629 | 0.517 | 0.742 | 0.832 |
|  |  | **BARCELONA AND MADRID (SPANISH DATA APPLIED)** | | | | | | |  |  |
| Low | Current | 0.592 | 0.470 | 0.295 | 0.161 |  | 0.453 | 0.173 | 0.040 | 0.009 |
|  | Former | 0.172 | 0.278 | 0.409 | 0.530 |  | 0.141 | 0.074 | 0.034 | 0.016 |
|  | Never | 0.236 | 0.252 | 0.295 | 0.309 |  | 0.406 | 0.753 | 0.926 | 0.975 |
| Middle | Current | 0.514 | 0.471 | 0.317 | 0.171 |  | 0.461 | 0.353 | 0.129 | 0.030 |
|  | Former | 0.197 | 0.261 | 0.419 | 0.616 |  | 0.181 | 0.173 | 0.121 | 0.073 |
|  | Never | 0.289 | 0.268 | 0.264 | 0.214 |  | 0.359 | 0.475 | 0.751 | 0.898 |
| High | Current | 0.398 | 0.359 | 0.274 | 0.192 |  | 0.349 | 0.395 | 0.167 | 0.033 |
|  | Former | 0.192 | 0.351 | 0.476 | 0.545 |  | 0.209 | 0.233 | 0.175 | 0.107 |
|  | Never | 0.410 | 0.289 | 0.249 | 0.263 |  | 0.443 | 0.372 | 0.657 | 0.860 |
|  |  | **BASQUE COUNTRY** | | |  |  |  |  |  |  |
| Low | Current | 0.533 | 0.420 | 0.288 | 0.188 |  | 0.460 | 0.182 | 0.064 | 0.027 |
|  | Former | 0.159 | 0.243 | 0.317 | 0.370 |  | 0.131 | 0.093 | 0.051 | 0.025 |
|  | Never | 0.309 | 0.338 | 0.394 | 0.443 |  | 0.410 | 0.725 | 0.885 | 0.948 |
| Middle | Current | 0.476 | 0.370 | 0.224 | 0.119 |  | 0.381 | 0.287 | 0.148 | 0.063 |
|  | Former | 0.145 | 0.311 | 0.433 | 0.484 |  | 0.221 | 0.156 | 0.146 | 0.162 |
|  | Never | 0.379 | 0.318 | 0.343 | 0.398 |  | 0.398 | 0.557 | 0.707 | 0.775 |
| High | Current | 0.347 | 0.316 | 0.249 | 0.184 |  | 0.318 | 0.272 | 0.138 | 0.052 |
|  | Former | 0.176 | 0.325 | 0.403 | 0.410 |  | 0.193 | 0.210 | 0.139 | 0.071 |
|  | Never | 0.476 | 0.359 | 0.348 | 0.406 |  | 0.490 | 0.518 | 0.722 | 0.877 |
|  |  | **BELGIUM** | |  |  |  |  |  |  |  |
| Low | Current | 0.551 | 0.426 | 0.291 | 0.190 |  | 0.423 | 0.273 | 0.138 | 0.065 |
|  | Former | 0.203 | 0.352 | 0.495 | 0.595 |  | 0.182 | 0.234 | 0.243 | 0.225 |
|  | Never | 0.247 | 0.221 | 0.215 | 0.216 |  | 0.395 | 0.493 | 0.619 | 0.711 |
| Middle | Current | 0.417 | 0.382 | 0.278 | 0.177 |  | 0.338 | 0.271 | 0.163 | 0.086 |
|  | Former | 0.245 | 0.391 | 0.516 | 0.599 |  | 0.249 | 0.304 | 0.319 | 0.308 |
|  | Never | 0.338 | 0.227 | 0.206 | 0.224 |  | 0.413 | 0.426 | 0.518 | 0.606 |
| High | Current | 0.301 | 0.311 | 0.255 | 0.186 |  | 0.224 | 0.250 | 0.157 | 0.071 |
|  | Former | 0.242 | 0.437 | 0.547 | 0.581 |  | 0.275 | 0.334 | 0.355 | 0.349 |
|  | Never | 0.457 | 0.253 | 0.198 | 0.234 |  | 0.501 | 0.416 | 0.489 | 0.581 |
|  |  | **CZECH REPUBLIC** | |  |  |  |  |  |  |  |
| Low | Current | 0.613 | 0.453 | 0.280 | 0.162 |  | 0.463 | 0.346 | 0.120 | 0.026 |
|  | Former | 0.176 | 0.371 | 0.428 | 0.374 |  | 0.147 | 0.223 | 0.171 | 0.089 |
|  | Never | 0.211 | 0.177 | 0.292 | 0.463 |  | 0.391 | 0.431 | 0.709 | 0.885 |
| Middle | Current | 0.392 | 0.288 | 0.193 | 0.129 |  | 0.254 | 0.302 | 0.199 | 0.091 |
|  | Former | 0.187 | 0.356 | 0.384 | 0.313 |  | 0.124 | 0.187 | 0.219 | 0.221 |
|  | Never | 0.421 | 0.356 | 0.423 | 0.559 |  | 0.622 | 0.511 | 0.582 | 0.688 |
| High | Current | 0.239 | 0.256 | 0.164 | 0.078 |  | 0.216 | 0.161 | 0.088 | 0.043 |
|  | Former | 0.182 | 0.354 | 0.342 | 0.229 |  | 0.225 | 0.287 | 0.345 | 0.391 |
|  | Never | 0.579 | 0.390 | 0.494 | 0.693 |  | 0.559 | 0.552 | 0.566 | 0.565 |
|  |  | **DENMARK** | |  |  |  |  |  |  |  |
| Low | Current | 0.570 | 0.548 | 0.479 | 0.395 |  | 0.560 | 0.498 | 0.386 | 0.276 |
|  | Former | 0.160 | 0.260 | 0.361 | 0.439 |  | 0.149 | 0.209 | 0.246 | 0.259 |
|  | Never | 0.270 | 0.192 | 0.160 | 0.166 |  | 0.291 | 0.293 | 0.368 | 0.465 |
| Middle | Current | 0.455 | 0.466 | 0.413 | 0.336 |  | 0.461 | 0.426 | 0.342 | 0.253 |
|  | Former | 0.195 | 0.292 | 0.413 | 0.526 |  | 0.181 | 0.212 | 0.262 | 0.317 |
|  | Never | 0.351 | 0.241 | 0.174 | 0.138 |  | 0.358 | 0.362 | 0.397 | 0.430 |
| High | Current | 0.327 | 0.367 | 0.346 | 0.295 |  | 0.326 | 0.325 | 0.295 | 0.256 |
|  | Former | 0.191 | 0.308 | 0.407 | 0.472 |  | 0.218 | 0.288 | 0.333 | 0.353 |
|  | Never | 0.483 | 0.325 | 0.248 | 0.233 |  | 0.456 | 0.387 | 0.373 | 0.391 |
|  |  | **ENGLAND & WALES** | | |  |  |  |  |  |  |
| Low | Current | 0.455 | 0.362 | 0.242 | 0.151 |  | 0.411 | 0.344 | 0.242 | 0.157 |
|  | Former | 0.188 | 0.383 | 0.556 | 0.660 |  | 0.200 | 0.292 | 0.356 | 0.387 |
|  | Never | 0.356 | 0.255 | 0.202 | 0.189 |  | 0.389 | 0.363 | 0.402 | 0.456 |
| Middle | Current | 0.356 | 0.263 | 0.187 | 0.136 |  | 0.314 | 0.226 | 0.148 | 0.098 |
|  | Former | 0.238 | 0.386 | 0.508 | 0.586 |  | 0.252 | 0.297 | 0.351 | 0.401 |
|  | Never | 0.405 | 0.351 | 0.305 | 0.278 |  | 0.434 | 0.477 | 0.501 | 0.501 |
| High | Current | 0.224 | 0.125 | 0.094 | 0.089 |  | 0.182 | 0.174 | 0.130 | 0.087 |
|  | Former | 0.256 | 0.424 | 0.527 | 0.569 |  | 0.241 | 0.336 | 0.394 | 0.416 |
|  | Never | 0.520 | 0.451 | 0.379 | 0.342 |  | 0.578 | 0.491 | 0.477 | 0.497 |
|  |  | **ESTONIA** | |  |  |  |  |  |  |  |
| Low | Current | 0.659 | 0.580 | 0.456 | 0.335 |  | 0.405 | 0.274 | 0.126 | 0.049 |
|  | Former | 0.156 | 0.235 | 0.301 | 0.344 |  | 0.171 | 0.155 | 0.063 | 0.017 |
|  | Never | 0.185 | 0.186 | 0.243 | 0.321 |  | 0.424 | 0.571 | 0.811 | 0.933 |
| Middle | Current | 0.630 | 0.567 | 0.512 | 0.471 |  | 0.305 | 0.281 | 0.129 | 0.039 |
|  | Former | 0.219 | 0.307 | 0.171 | 0.052 |  | 0.166 | 0.157 | 0.177 | 0.212 |
|  | Never | 0.151 | 0.127 | 0.317 | 0.477 |  | 0.529 | 0.562 | 0.694 | 0.748 |
| High | Current | 0.337 | 0.375 | 0.333 | 0.261 |  | 0.159 | 0.234 | 0.102 | 0.021 |
|  | Former | 0.253 | 0.250 | 0.407 | 0.656 |  | 0.181 | 0.201 | 0.122 | 0.054 |
|  | Never | 0.411 | 0.375 | 0.259 | 0.082 |  | 0.659 | 0.565 | 0.776 | 0.925 |
|  |  | **FINLAND** | |  |  |  |  |  |  |  |
| Low | Current | 0.491 | 0.365 | 0.273 | 0.215 |  | 0.436 | 0.261 | 0.119 | 0.052 |
|  | Former | 0.209 | 0.341 | 0.384 | 0.357 |  | 0.152 | 0.172 | 0.103 | 0.044 |
|  | Never | 0.300 | 0.294 | 0.344 | 0.428 |  | 0.412 | 0.567 | 0.778 | 0.905 |
| Middle | Current | 0.438 | 0.332 | 0.165 | 0.065 |  | 0.308 | 0.214 | 0.095 | 0.035 |
|  | Former | 0.224 | 0.359 | 0.468 | 0.534 |  | 0.185 | 0.194 | 0.150 | 0.100 |
|  | Never | 0.338 | 0.309 | 0.367 | 0.401 |  | 0.507 | 0.592 | 0.755 | 0.865 |
| High | Current | 0.258 | 0.244 | 0.155 | 0.080 |  | 0.187 | 0.152 | 0.101 | 0.063 |
|  | Former | 0.166 | 0.252 | 0.429 | 0.635 |  | 0.141 | 0.181 | 0.116 | 0.051 |
|  | Never | 0.576 | 0.505 | 0.417 | 0.286 |  | 0.673 | 0.667 | 0.783 | 0.886 |
|  |  | **FRANCE** |  |  |  |  |  |  |  |  |
| Low | Current | 0.536 | 0.341 | 0.208 | 0.137 |  | 0.399 | 0.209 | 0.082 | 0.031 |
|  | Former | 0.172 | 0.258 | 0.312 | 0.332 |  | 0.121 | 0.093 | 0.068 | 0.050 |
|  | Never | 0.292 | 0.402 | 0.480 | 0.531 |  | 0.480 | 0.698 | 0.850 | 0.919 |
| Middle | Current | 0.446 | 0.286 | 0.160 | 0.089 |  | 0.379 | 0.224 | 0.117 | 0.063 |
|  | Former | 0.183 | 0.320 | 0.399 | 0.418 |  | 0.151 | 0.112 | 0.092 | 0.081 |
|  | Never | 0.371 | 0.394 | 0.441 | 0.493 |  | 0.470 | 0.664 | 0.791 | 0.856 |
| High | Current | 0.278 | 0.262 | 0.193 | 0.125 |  | 0.245 | 0.212 | 0.137 | 0.077 |
|  | Former | 0.143 | 0.310 | 0.384 | 0.363 |  | 0.171 | 0.171 | 0.136 | 0.098 |
|  | Never | 0.579 | 0.428 | 0.424 | 0.512 |  | 0.584 | 0.617 | 0.727 | 0.825 |
|  |  | **LITHUANIA** | |  |  |  |  |  |  |  |
| Low | Current | 0.708 | 0.543 | 0.393 | 0.290 |  | 0.322 | 0.074 | 0.033 | 0.026 |
|  | Former | 0.088 | 0.163 | 0.218 | 0.239 |  | 0.051 | 0.018 | 0.020 | 0.041 |
|  | Never | 0.204 | 0.294 | 0.390 | 0.471 |  | 0.627 | 0.908 | 0.947 | 0.932 |
| Middle | Current | 0.597 | 0.505 | 0.294 | 0.127 |  | 0.221 | 0.119 | 0.023 | 0.003 |
|  | Former | 0.153 | 0.215 | 0.279 | 0.335 |  | 0.058 | 0.038 | 0.023 | 0.015 |
|  | Never | 0.250 | 0.280 | 0.427 | 0.538 |  | 0.721 | 0.843 | 0.953 | 0.982 |
| High | Current | 0.467 | 0.376 | 0.267 | 0.181 |  | 0.208 | 0.141 | 0.043 | 0.009 |
|  | Former | 0.115 | 0.218 | 0.317 | 0.383 |  | 0.045 | 0.058 | 0.029 | 0.009 |
|  | Never | 0.418 | 0.406 | 0.417 | 0.436 |  | 0.747 | 0.802 | 0.929 | 0.982 |
|  |  | **NETHERLANDS** | |  |  |  |  |  |  |  |
| Low | Current | 0.536 | 0.448 | 0.347 | 0.263 |  | 0.434 | 0.339 | 0.231 | 0.150 |
|  | Former | 0.226 | 0.359 | 0.506 | 0.625 |  | 0.246 | 0.361 | 0.374 | 0.324 |
|  | Never | 0.238 | 0.194 | 0.148 | 0.112 |  | 0.320 | 0.300 | 0.395 | 0.526 |
| Middle | Current | 0.408 | 0.359 | 0.282 | 0.212 |  | 0.286 | 0.286 | 0.222 | 0.151 |
|  | Former | 0.234 | 0.434 | 0.588 | 0.674 |  | 0.295 | 0.408 | 0.442 | 0.424 |
|  | Never | 0.358 | 0.207 | 0.129 | 0.115 |  | 0.419 | 0.306 | 0.335 | 0.425 |
| High | Current | 0.300 | 0.287 | 0.253 | 0.215 |  | 0.234 | 0.216 | 0.190 | 0.165 |
|  | Former | 0.216 | 0.400 | 0.560 | 0.661 |  | 0.270 | 0.442 | 0.458 | 0.377 |
|  | Never | 0.485 | 0.313 | 0.187 | 0.124 |  | 0.496 | 0.342 | 0.353 | 0.458 |
|  |  | **NORWAY** | |  |  |  |  |  |  |  |
| Low | Current | 0.465 | 0.428 | 0.289 | 0.159 |  | 0.552 | 0.403 | 0.259 | 0.162 |
|  | Former | 0.228 | 0.321 | 0.459 | 0.596 |  | 0.191 | 0.278 | 0.293 | 0.260 |
|  | Never | 0.307 | 0.251 | 0.252 | 0.245 |  | 0.258 | 0.320 | 0.448 | 0.578 |
| Middle | Current | 0.332 | 0.338 | 0.339 | 0.337 |  | 0.333 | 0.304 | 0.232 | 0.163 |
|  | Former | 0.200 | 0.369 | 0.436 | 0.419 |  | 0.245 | 0.334 | 0.288 | 0.191 |
|  | Never | 0.468 | 0.294 | 0.225 | 0.245 |  | 0.422 | 0.362 | 0.480 | 0.646 |
| High | Current | 0.139 | 0.184 | 0.132 | 0.067 |  | 0.186 | 0.213 | 0.176 | 0.123 |
|  | Former | 0.235 | 0.311 | 0.384 | 0.443 |  | 0.208 | 0.360 | 0.118 | 0.012 |
|  | Never | 0.627 | 0.505 | 0.484 | 0.490 |  | 0.606 | 0.427 | 0.706 | 0.866 |
|  |  | **POLAND** | |  |  |  |  |  |  |  |
| Low | Current | 0.612 | 0.574 | 0.387 | 0.193 |  | 0.490 | 0.354 | 0.144 | 0.042 |
|  | Former | 0.131 | 0.234 | 0.373 | 0.508 |  | 0.107 | 0.170 | 0.132 | 0.069 |
|  | Never | 0.257 | 0.192 | 0.240 | 0.300 |  | 0.403 | 0.476 | 0.724 | 0.889 |
| Middle | Current | 0.457 | 0.455 | 0.320 | 0.174 |  | 0.322 | 0.342 | 0.201 | 0.080 |
|  | Former | 0.173 | 0.288 | 0.409 | 0.506 |  | 0.131 | 0.186 | 0.210 | 0.207 |
|  | Never | 0.370 | 0.256 | 0.271 | 0.320 |  | 0.548 | 0.472 | 0.589 | 0.713 |
| High | Current | 0.250 | 0.303 | 0.175 | 0.063 |  | 0.196 | 0.264 | 0.210 | 0.123 |
|  | Former | 0.177 | 0.331 | 0.470 | 0.561 |  | 0.119 | 0.221 | 0.251 | 0.219 |
|  | Never | 0.573 | 0.366 | 0.355 | 0.376 |  | 0.686 | 0.515 | 0.539 | 0.658 |
|  |  | **SCOTLAND** | |  |  |  |  |  |  |  |
| Low | Current | 0.604 | 0.406 | 0.275 | 0.204 |  | 0.560 | 0.462 | 0.317 | 0.196 |
|  | Former | 0.164 | 0.353 | 0.508 | 0.590 |  | 0.168 | 0.292 | 0.369 | 0.391 |
|  | Never | 0.232 | 0.241 | 0.217 | 0.206 |  | 0.271 | 0.246 | 0.314 | 0.413 |
| Middle | Current | 0.378 | 0.318 | 0.217 | 0.135 |  | 0.367 | 0.265 | 0.190 | 0.144 |
|  | Former | 0.267 | 0.418 | 0.545 | 0.630 |  | 0.226 | 0.323 | 0.361 | 0.353 |
|  | Never | 0.355 | 0.265 | 0.237 | 0.236 |  | 0.407 | 0.412 | 0.449 | 0.503 |
| High | Current | 0.195 | 0.153 | 0.109 | 0.076 |  | 0.199 | 0.179 | 0.121 | 0.071 |
|  | Former | 0.307 | 0.483 | 0.603 | 0.666 |  | 0.274 | 0.370 | 0.433 | 0.464 |
|  | Never | 0.498 | 0.365 | 0.289 | 0.258 |  | 0.527 | 0.451 | 0.447 | 0.465 |
|  |  | **SWEDEN** | |  |  |  |  |  |  |  |
| Low | Current | 0.345 | 0.279 | 0.195 | 0.130 |  | 0.472 | 0.348 | 0.217 | 0.129 |
|  | Former | 0.285 | 0.437 | 0.527 | 0.562 |  | 0.198 | 0.269 | 0.260 | 0.209 |
|  | Never | 0.370 | 0.284 | 0.278 | 0.308 |  | 0.330 | 0.383 | 0.523 | 0.662 |
| Middle | Current | 0.179 | 0.266 | 0.199 | 0.099 |  | 0.260 | 0.307 | 0.192 | 0.081 |
|  | Former | 0.269 | 0.444 | 0.521 | 0.522 |  | 0.305 | 0.324 | 0.315 | 0.292 |
|  | Never | 0.553 | 0.290 | 0.280 | 0.379 |  | 0.435 | 0.369 | 0.493 | 0.627 |
| High | Current | 0.090 | 0.131 | 0.113 | 0.074 |  | 0.116 | 0.181 | 0.139 | 0.073 |
|  | Former | 0.245 | 0.419 | 0.511 | 0.532 |  | 0.234 | 0.333 | 0.356 | 0.327 |
|  | Never | 0.664 | 0.450 | 0.376 | 0.394 |  | 0.650 | 0.485 | 0.504 | 0.600 |
|  |  | **SWITZERLAND** | |  |  |  |  |  |  |  |
| Low | Current | 0.466 | 0.438 | 0.341 | 0.237 |  | 0.409 | 0.269 | 0.159 | 0.095 |
|  | Former | 0.195 | 0.307 | 0.380 | 0.410 |  | 0.138 | 0.175 | 0.162 | 0.126 |
|  | Never | 0.340 | 0.255 | 0.279 | 0.354 |  | 0.453 | 0.555 | 0.679 | 0.779 |
| Middle | Current | 0.424 | 0.367 | 0.272 | 0.188 |  | 0.317 | 0.269 | 0.162 | 0.082 |
|  | Former | 0.165 | 0.316 | 0.417 | 0.454 |  | 0.167 | 0.223 | 0.220 | 0.184 |
|  | Never | 0.411 | 0.317 | 0.311 | 0.358 |  | 0.516 | 0.508 | 0.619 | 0.734 |
| High | Current | 0.336 | 0.325 | 0.265 | 0.199 |  | 0.242 | 0.243 | 0.217 | 0.183 |
|  | Former | 0.182 | 0.329 | 0.410 | 0.423 |  | 0.204 | 0.250 | 0.221 | 0.164 |
|  | Never | 0.483 | 0.347 | 0.325 | 0.377 |  | 0.554 | 0.506 | 0.561 | 0.652 |
|  |  | **TURIN AND TUSCANY (ITALIAN DATA APPLIED)** | | | | | | |  |  |
| Low | Current | 0.462 | 0.353 | 0.241 | 0.161 |  | 0.281 | 0.180 | 0.090 | 0.042 |
|  | Former | 0.199 | 0.338 | 0.445 | 0.504 |  | 0.138 | 0.120 | 0.102 | 0.089 |
|  | Never | 0.339 | 0.309 | 0.314 | 0.335 |  | 0.581 | 0.701 | 0.808 | 0.869 |
| Middle | Current | 0.342 | 0.330 | 0.248 | 0.163 |  | 0.249 | 0.255 | 0.197 | 0.131 |
|  | Former | 0.192 | 0.358 | 0.486 | 0.553 |  | 0.184 | 0.199 | 0.196 | 0.184 |
|  | Never | 0.466 | 0.312 | 0.266 | 0.284 |  | 0.567 | 0.545 | 0.607 | 0.686 |
| High | Current | 0.267 | 0.318 | 0.248 | 0.150 |  | 0.212 | 0.271 | 0.212 | 0.125 |
|  | Former | 0.168 | 0.319 | 0.453 | 0.536 |  | 0.180 | 0.215 | 0.233 | 0.237 |
|  | Never | 0.565 | 0.363 | 0.299 | 0.314 |  | 0.608 | 0.514 | 0.556 | 0.638 |

Self-reported smoking behaviour classified as ‘current’ (including both regular and occasional smokers), ‘former’ or ‘never’.

**Table S6: Prevalences of overweight**

|  |  | Men | | | |  | Women | | | |
| --- | --- | --- | --- | --- | --- | --- | --- | --- | --- | --- |
|  | **age** | 30-44 | 45-59 | 60-69 | 70-79 |  | 30-44 | 45-59 | 60-69 | 70-79 |
| Education | BMI | **AUSTRIA** | | | | | | |  |  |
| Low | 30+ | 0.129 | 0.222 | 0.222 | 0.167 |  | 0.093 | 0.183 | 0.198 | 0.155 |
|  | 25-30 | 0.469 | 0.495 | 0.486 | 0.460 |  | 0.301 | 0.441 | 0.487 | 0.469 |
|  | -25 | 0.402 | 0.284 | 0.292 | 0.373 |  | 0.607 | 0.376 | 0.315 | 0.376 |
| Middle | 30+ | 0.086 | 0.177 | 0.203 | 0.171 |  | 0.044 | 0.126 | 0.139 | 0.093 |
|  | 25-30 | 0.415 | 0.512 | 0.500 | 0.429 |  | 0.178 | 0.328 | 0.413 | 0.428 |
|  | -25 | 0.500 | 0.312 | 0.297 | 0.400 |  | 0.778 | 0.546 | 0.448 | 0.479 |
| High | 30+ | 0.013 | 0.047 | 0.077 | 0.077 |  | 0.017 | 0.048 | 0.063 | 0.053 |
|  | 25-30 | 0.314 | 0.461 | 0.521 | 0.520 |  | 0.154 | 0.247 | 0.317 | 0.353 |
|  | -25 | 0.672 | 0.492 | 0.402 | 0.403 |  | 0.829 | 0.705 | 0.620 | 0.594 |
|  |  | **BARCELONA AND MADRID (SPANISH DATA APPLIED)** | | | | | | |  |  |
| Low | 30+ | 0.129 | 0.172 | 0.186 | 0.178 |  | 0.104 | 0.223 | 0.273 | 0.248 |
|  | 25-30 | 0.491 | 0.554 | 0.552 | 0.515 |  | 0.278 | 0.410 | 0.450 | 0.427 |
|  | -25 | 0.379 | 0.274 | 0.262 | 0.307 |  | 0.618 | 0.366 | 0.277 | 0.325 |
| Middle | 30+ | 0.088 | 0.139 | 0.128 | 0.087 |  | 0.051 | 0.076 | 0.099 | 0.114 |
|  | 25-30 | 0.499 | 0.541 | 0.530 | 0.492 |  | 0.166 | 0.300 | 0.375 | 0.387 |
|  | -25 | 0.414 | 0.320 | 0.342 | 0.421 |  | 0.783 | 0.623 | 0.526 | 0.499 |
| High | 30+ | 0.073 | 0.113 | 0.128 | 0.121 |  | 0.034 | 0.060 | 0.096 | 0.136 |
|  | 25-30 | 0.433 | 0.502 | 0.536 | 0.545 |  | 0.176 | 0.245 | 0.336 | 0.427 |
|  | -25 | 0.494 | 0.385 | 0.337 | 0.334 |  | 0.791 | 0.695 | 0.568 | 0.437 |
|  |  | **BASQUE COUNTRY** | |  |  |  |  |  |  |  |
| Low | 30+ | 0.146 | 0.163 | 0.156 | 0.139 |  | 0.067 | 0.140 | 0.187 | 0.190 |
|  | 25-30 | 0.449 | 0.545 | 0.572 | 0.559 |  | 0.265 | 0.395 | 0.435 | 0.411 |
|  | -25 | 0.405 | 0.292 | 0.271 | 0.303 |  | 0.669 | 0.465 | 0.379 | 0.399 |
| Middle | 30+ | 0.062 | 0.157 | 0.131 | 0.060 |  | 0.053 | 0.070 | 0.101 | 0.142 |
|  | 25-30 | 0.444 | 0.519 | 0.560 | 0.575 |  | 0.180 | 0.327 | 0.361 | 0.312 |
|  | -25 | 0.494 | 0.324 | 0.309 | 0.365 |  | 0.768 | 0.603 | 0.538 | 0.546 |
| High | 30+ | 0.072 | 0.126 | 0.127 | 0.096 |  | 0.035 | 0.063 | 0.082 | 0.087 |
|  | 25-30 | 0.444 | 0.548 | 0.594 | 0.603 |  | 0.153 | 0.239 | 0.262 | 0.238 |
|  | -25 | 0.484 | 0.327 | 0.279 | 0.301 |  | 0.812 | 0.698 | 0.656 | 0.676 |
|  |  | **BELGIUM** | | |  |  |  |  |  |  |
| Low | 30+ | 0.129 | 0.205 | 0.193 | 0.137 |  | 0.154 | 0.211 | 0.213 | 0.182 |
|  | 25-30 | 0.394 | 0.468 | 0.470 | 0.432 |  | 0.247 | 0.352 | 0.378 | 0.350 |
|  | -25 | 0.477 | 0.327 | 0.337 | 0.431 |  | 0.599 | 0.437 | 0.409 | 0.468 |
| Middle | 30+ | 0.120 | 0.162 | 0.156 | 0.125 |  | 0.077 | 0.136 | 0.143 | 0.112 |
|  | 25-30 | 0.384 | 0.463 | 0.478 | 0.455 |  | 0.228 | 0.292 | 0.332 | 0.350 |
|  | -25 | 0.497 | 0.375 | 0.365 | 0.420 |  | 0.695 | 0.571 | 0.525 | 0.539 |
| High | 30+ | 0.071 | 0.120 | 0.117 | 0.085 |  | 0.043 | 0.080 | 0.099 | 0.096 |
|  | 25-30 | 0.339 | 0.453 | 0.467 | 0.421 |  | 0.136 | 0.243 | 0.297 | 0.296 |
|  | -25 | 0.590 | 0.427 | 0.417 | 0.494 |  | 0.821 | 0.677 | 0.604 | 0.608 |
|  |  | **CZECH REPUBLIC** | |  |  |  |  |  |  |  |
| Low | 30+ | 0.142 | 0.221 | 0.244 | 0.223 |  | 0.148 | 0.301 | 0.337 | 0.280 |
|  | 25-30 | 0.397 | 0.514 | 0.527 | 0.480 |  | 0.258 | 0.400 | 0.441 | 0.413 |
|  | -25 | 0.461 | 0.265 | 0.229 | 0.297 |  | 0.595 | 0.299 | 0.221 | 0.307 |
| Middle | 30+ | 0.029 | 0.213 | 0.179 | 0.046 |  | 0.084 | 0.167 | 0.148 | 0.085 |
|  | 25-30 | 0.596 | 0.539 | 0.514 | 0.509 |  | 0.182 | 0.441 | 0.525 | 0.470 |
|  | -25 | 0.375 | 0.248 | 0.307 | 0.445 |  | 0.735 | 0.392 | 0.327 | 0.446 |
| High | 30+ | 0.109 | 0.199 | 0.137 | 0.054 |  | 0.029 | 0.107 | 0.156 | 0.136 |
|  | 25-30 | 0.399 | 0.629 | 0.613 | 0.463 |  | 0.191 | 0.539 | 0.582 | 0.426 |
|  | -25 | 0.493 | 0.172 | 0.250 | 0.483 |  | 0.781 | 0.354 | 0.262 | 0.438 |
|  |  | **DENMARK** | |  |  |  |  |  |  |  |
| Low | 30+ | 0.145 | 0.216 | 0.177 | 0.103 |  | 0.134 | 0.171 | 0.160 | 0.127 |
|  | 25-30 | 0.434 | 0.500 | 0.502 | 0.468 |  | 0.271 | 0.356 | 0.378 | 0.357 |
|  | -25 | 0.421 | 0.284 | 0.322 | 0.429 |  | 0.595 | 0.473 | 0.463 | 0.516 |
| Middle | 30+ | 0.098 | 0.151 | 0.141 | 0.101 |  | 0.138 | 0.108 | 0.101 | 0.105 |
|  | 25-30 | 0.405 | 0.500 | 0.518 | 0.491 |  | 0.207 | 0.308 | 0.337 | 0.316 |
|  | -25 | 0.497 | 0.349 | 0.341 | 0.408 |  | 0.655 | 0.584 | 0.561 | 0.579 |
| High | 30+ | 0.066 | 0.101 | 0.097 | 0.072 |  | 0.075 | 0.075 | 0.075 | 0.075 |
|  | 25-30 | 0.394 | 0.454 | 0.452 | 0.415 |  | 0.210 | 0.266 | 0.288 | 0.285 |
|  | -25 | 0.540 | 0.445 | 0.452 | 0.514 |  | 0.715 | 0.659 | 0.637 | 0.640 |
|  |  | **ENGLAND & WALES** | |  |  |  |  |  |  |  |
| Low | 30+ | 0.249 | 0.310 | 0.303 | 0.258 |  | 0.286 | 0.359 | 0.352 | 0.302 |
|  | 25-30 | 0.446 | 0.453 | 0.464 | 0.475 |  | 0.285 | 0.359 | 0.385 | 0.378 |
|  | -25 | 0.306 | 0.237 | 0.234 | 0.266 |  | 0.429 | 0.282 | 0.264 | 0.321 |
| Middle | 30+ | 0.212 | 0.251 | 0.242 | 0.210 |  | 0.228 | 0.263 | 0.258 | 0.232 |
|  | 25-30 | 0.472 | 0.519 | 0.523 | 0.504 |  | 0.309 | 0.361 | 0.382 | 0.382 |
|  | -25 | 0.316 | 0.231 | 0.235 | 0.286 |  | 0.463 | 0.376 | 0.360 | 0.386 |
| High | 30+ | 0.181 | 0.219 | 0.206 | 0.169 |  | 0.170 | 0.226 | 0.224 | 0.189 |
|  | 25-30 | 0.498 | 0.544 | 0.570 | 0.581 |  | 0.254 | 0.335 | 0.388 | 0.414 |
|  | -25 | 0.322 | 0.237 | 0.224 | 0.250 |  | 0.576 | 0.439 | 0.388 | 0.397 |
|  |  | **ESTONIA** | | |  |  |  |  |  |  |
| Low | 30+ | 0.167 | 0.216 | 0.245 | 0.256* |  | 0.158 | 0.259 | 0.391 | 0.516* |
|  | 25-30 | 0.338 | 0.413 | 0.321 | 0.185* |  | 0.294 | 0.362 | 0.336 | 0.267* |
|  | -25 | 0.495 | 0.371 | 0.434 | 0.559* |  | 0.548 | 0.379 | 0.273 | 0.217* |
| Middle | 30+ | 0.136 | 0.156 | 0.167 | 0.170* |  | 0.114 | 0.203 | 0.284 | 0.338* |
|  | 25-30 | 0.395 | 0.429 | 0.429 | 0.410* |  | 0.229 | 0.392 | 0.409 | 0.334* |
|  | -25 | 0.469 | 0.416 | 0.405 | 0.420* |  | 0.657 | 0.406 | 0.307 | 0.328* |
| High | 30+ | 0.126 | 0.188 | 0.250 | 0.302* |  | 0.063 | 0.145 | 0.241 | 0.317* |
|  | 25-30 | 0.432 | 0.475 | 0.357 | 0.203* |  | 0.169 | 0.384 | 0.407 | 0.300* |
|  | -25 | 0.442 | 0.338 | 0.393 | 0.495* |  | 0.767 | 0.472 | 0.352 | 0.383* |
|  |  | **FINLAND** | |  |  |  |  |  |  |  |
| Low | 30+ | 0.121 | 0.198 | 0.180 | 0.120* |  | 0.105 | 0.199 | 0.218 | 0.179* |
|  | 25-30 | 0.433 | 0.486 | 0.530 | 0.563* |  | 0.284 | 0.357 | 0.430 | 0.491* |
|  | -25 | 0.446 | 0.316 | 0.290 | 0.317* |  | 0.612 | 0.445 | 0.352 | 0.330* |
| Middle | 30+ | 0.112 | 0.140 | 0.165 | 0.184* |  | 0.097 | 0.136 | 0.225 | 0.358* |
|  | 25-30 | 0.422 | 0.501 | 0.534 | 0.538* |  | 0.245 | 0.356 | 0.402 | 0.397* |
|  | -25 | 0.466 | 0.360 | 0.301 | 0.279* |  | 0.658 | 0.508 | 0.373 | 0.245* |
| High | 30+ | 0.078 | 0.112 | 0.212 | 0.381* |  | 0.083 | 0.113 | 0.151 | 0.189* |
|  | 25-30 | 0.426 | 0.508 | 0.447 | 0.324* |  | 0.177 | 0.298 | 0.315 | 0.261* |
|  | -25 | 0.497 | 0.380 | 0.341 | 0.295* |  | 0.740 | 0.588 | 0.534 | 0.550* |
|  |  | **FRANCE** | |  |  |  |  |  |  |  |
| Low | 30+ | 0.108 | 0.189 | 0.197 | 0.157 |  | 0.143 | 0.192 | 0.193 | 0.166 |
|  | 25-30 | 0.390 | 0.459 | 0.495 | 0.505 |  | 0.238 | 0.306 | 0.343 | 0.355 |
|  | -25 | 0.502 | 0.352 | 0.308 | 0.338 |  | 0.619 | 0.502 | 0.464 | 0.479 |
| Middle | 30+ | 0.079 | 0.140 | 0.156 | 0.134 |  | 0.086 | 0.113 | 0.127 | 0.130 |
|  | 25-30 | 0.379 | 0.480 | 0.517 | 0.512 |  | 0.182 | 0.232 | 0.271 | 0.296 |
|  | -25 | 0.542 | 0.381 | 0.328 | 0.354 |  | 0.731 | 0.656 | 0.602 | 0.574 |
| High | 30+ | 0.050 | 0.093 | 0.096 | 0.073 |  | 0.036 | 0.069 | 0.080 | 0.071 |
|  | 25-30 | 0.312 | 0.417 | 0.462 | 0.465 |  | 0.139 | 0.177 | 0.207 | 0.228 |
|  | -25 | 0.638 | 0.490 | 0.441 | 0.463 |  | 0.824 | 0.754 | 0.713 | 0.702 |
|  |  | **HUNGARY** | |  |  |  |  |  |  |  |
| Low | 30+ | 0.201 | 0.272 | 0.266 | 0.218 |  | 0.211 | 0.316 | 0.309 | 0.241 |
|  | 25-30 | 0.372 | 0.368 | 0.389 | 0.422 |  | 0.250 | 0.361 | 0.385 | 0.352 |
|  | -25 | 0.427 | 0.360 | 0.345 | 0.360 |  | 0.539 | 0.323 | 0.306 | 0.407 |
| Middle | 30+ | 0.179 | 0.257 | 0.261 | 0.220 |  | 0.171 | 0.253 | 0.250 | 0.199 |
|  | 25-30 | 0.431 | 0.422 | 0.423 | 0.429 |  | 0.245 | 0.380 | 0.442 | 0.444 |
|  | -25 | 0.390 | 0.322 | 0.317 | 0.351 |  | 0.584 | 0.367 | 0.309 | 0.357 |
| High | 30+ | 0.184 | 0.209 | 0.187 | 0.149 |  | 0.067 | 0.152 | 0.192 | 0.177 |
|  | 25-30 | 0.419 | 0.496 | 0.495 | 0.451 |  | 0.257 | 0.349 | 0.382 | 0.372 |
|  | -25 | 0.397 | 0.295 | 0.318 | 0.400 |  | 0.676 | 0.499 | 0.426 | 0.451 |
|  |  | **LITHUANIA** | |  |  |  |  |  |  |  |
| Low | 30+ | 0.086 | 0.184 | 0.199 | 0.149* |  | 0.173 | 0.315 | 0.336 | 0.273 |
|  | 25-30 | 0.404 | 0.413 | 0.407 | 0.394* |  | 0.290 | 0.410 | 0.397 | 0.312 |
|  | -25 | 0.510 | 0.403 | 0.394 | 0.457* |  | 0.537 | 0.275 | 0.268 | 0.415 |
| Middle | 30+ | 0.102 | 0.177 | 0.214 | 0.212* |  | 0.120 | 0.256 | 0.322 | 0.309 |
|  | 25-30 | 0.399 | 0.448 | 0.414 | 0.343* |  | 0.294 | 0.387 | 0.444 | 0.471 |
|  | -25 | 0.499 | 0.376 | 0.371 | 0.445* |  | 0.586 | 0.357 | 0.233 | 0.220 |
| High | 30+ | 0.102 | 0.207 | 0.164 | 0.077* |  | 0.089 | 0.211 | 0.357 | 0.471* |
|  | 25-30 | 0.474 | 0.531 | 0.459 | 0.333* |  | 0.225 | 0.395 | 0.314 | 0.152* |
|  | -25 | 0.424 | 0.262 | 0.377 | 0.590* |  | 0.686 | 0.395 | 0.329 | 0.377* |
|  |  | **NETHERLANDS** | |  |  |  |  |  |  |  |
| Low | 30+ | 0.159 | 0.177 | 0.151 | 0.113 |  | 0.149 | 0.169 | 0.166 | 0.151 |
|  | 25-30 | 0.370 | 0.457 | 0.476 | 0.452 |  | 0.307 | 0.387 | 0.424 | 0.429 |
|  | -25 | 0.470 | 0.366 | 0.373 | 0.435 |  | 0.544 | 0.444 | 0.410 | 0.419 |
| Middle | 30+ | 0.092 | 0.114 | 0.106 | 0.085 |  | 0.100 | 0.137 | 0.124 | 0.090 |
|  | 25-30 | 0.342 | 0.431 | 0.452 | 0.430 |  | 0.264 | 0.328 | 0.383 | 0.424 |
|  | -25 | 0.566 | 0.455 | 0.442 | 0.485 |  | 0.636 | 0.535 | 0.493 | 0.486 |
| High | 30+ | 0.047 | 0.090 | 0.093 | 0.070 |  | 0.072 | 0.077 | 0.087 | 0.100 |
|  | 25-30 | 0.326 | 0.394 | 0.411 | 0.395 |  | 0.188 | 0.271 | 0.315 | 0.325 |
|  | -25 | 0.627 | 0.516 | 0.496 | 0.535 |  | 0.740 | 0.652 | 0.598 | 0.575 |
|  |  | **NORWAY** | |  |  |  |  |  |  |  |
| Low | 30+ | 0.137 | 0.138 | 0.106 | 0.073 |  | 0.067 | 0.123 | 0.142 | 0.125 |
|  | 25-30 | 0.428 | 0.416 | 0.420 | 0.432 |  | 0.290 | 0.359 | 0.386 | 0.383 |
|  | -25 | 0.434 | 0.446 | 0.474 | 0.495 |  | 0.643 | 0.518 | 0.473 | 0.492 |
| Middle | 30+ | 0.140 | 0.111 | 0.072 | 0.045 |  | 0.067 | 0.103 | 0.107 | 0.089 |
|  | 25-30 | 0.461 | 0.520 | 0.518 | 0.484 |  | 0.276 | 0.325 | 0.353 | 0.363 |
|  | -25 | 0.399 | 0.370 | 0.410 | 0.471 |  | 0.657 | 0.572 | 0.540 | 0.548 |
| High | 30+ | 0.056 | 0.073 | 0.044 | 0.018 |  | 0.055 | 0.087 | 0.073 | 0.044 |
|  | 25-30 | 0.470 | 0.484 | 0.407 | 0.301 |  | 0.210 | 0.245 | 0.255 | 0.248 |
|  | -25 | 0.474 | 0.443 | 0.549 | 0.681 |  | 0.735 | 0.668 | 0.672 | 0.708 |
|  |  | **POLAND** | |  |  |  |  |  |  |  |
| Low | 30+ | 0.142 | 0.223 | 0.236 | 0.200 |  | 0.160 | 0.321 | 0.342 | 0.265 |
|  | 25-30 | 0.483 | 0.438 | 0.457 | 0.510 |  | 0.241 | 0.345 | 0.407 | 0.429 |
|  | -25 | 0.376 | 0.339 | 0.307 | 0.290 |  | 0.599 | 0.334 | 0.252 | 0.306 |
| Middle | 30+ | 0.168 | 0.241 | 0.261 | 0.243 |  | 0.093 | 0.214 | 0.281 | 0.274 |
|  | 25-30 | 0.519 | 0.496 | 0.508 | 0.537 |  | 0.284 | 0.377 | 0.411 | 0.404 |
|  | -25 | 0.314 | 0.264 | 0.232 | 0.220 |  | 0.623 | 0.409 | 0.308 | 0.323 |
| High | 30+ | 0.141 | 0.215 | 0.211 | 0.164 |  | 0.046 | 0.113 | 0.158 | 0.160 |
|  | 25-30 | 0.496 | 0.554 | 0.512 | 0.421 |  | 0.193 | 0.370 | 0.441 | 0.421 |
|  | -25 | 0.364 | 0.231 | 0.277 | 0.415 |  | 0.762 | 0.517 | 0.401 | 0.419 |
|  |  | **SCOTLAND** | |  |  |  |  |  |  |  |
| Low | 30+ | 0.168 | 0.329 | 0.319 | 0.231 |  | 0.243 | 0.304 | 0.302 | 0.263 |
|  | 25-30 | 0.273 | 0.371 | 0.387 | 0.353 |  | 0.284 | 0.328 | 0.310 | 0.263 |
|  | -25 | 0.559 | 0.301 | 0.294 | 0.417 |  | 0.473 | 0.369 | 0.388 | 0.474 |
| Middle | 30+ | 0.226 | 0.228 | 0.217 | 0.201 |  | 0.221 | 0.273 | 0.282 | 0.271 |
|  | 25-30 | 0.393 | 0.430 | 0.445 | 0.443 |  | 0.272 | 0.336 | 0.338 | 0.305 |
|  | -25 | 0.381 | 0.342 | 0.339 | 0.356 |  | 0.507 | 0.392 | 0.380 | 0.424 |
| High | 30+ | 0.164 | 0.231 | 0.218 | 0.163 |  | 0.139 | 0.233 | 0.247 | 0.215 |
|  | 25-30 | 0.426 | 0.421 | 0.425 | 0.435 |  | 0.260 | 0.296 | 0.334 | 0.367 |
|  | -25 | 0.409 | 0.349 | 0.357 | 0.403 |  | 0.601 | 0.471 | 0.419 | 0.418 |
|  |  | **SWEDEN** | |  |  |  |  |  |  |  |
| Low | 30+ | 0.107 | 0.150 | 0.152 | 0.127 |  | 0.125 | 0.177 | 0.188 | 0.171 |
|  | 25-30 | 0.487 | 0.486 | 0.471 | 0.451 |  | 0.322 | 0.347 | 0.365 | 0.378 |
|  | -25 | 0.406 | 0.364 | 0.377 | 0.422 |  | 0.553 | 0.477 | 0.447 | 0.451 |
| Middle | 30+ | 0.107 | 0.140 | 0.143 | 0.126 |  | 0.101 | 0.101 | 0.103 | 0.106 |
|  | 25-30 | 0.458 | 0.506 | 0.498 | 0.461 |  | 0.249 | 0.369 | 0.404 | 0.378 |
|  | -25 | 0.436 | 0.354 | 0.359 | 0.413 |  | 0.650 | 0.529 | 0.493 | 0.517 |
| High | 30+ | 0.047 | 0.085 | 0.092 | 0.075 |  | 0.052 | 0.095 | 0.118 | 0.116 |
|  | 25-30 | 0.392 | 0.474 | 0.485 | 0.456 |  | 0.207 | 0.278 | 0.299 | 0.284 |
|  | -25 | 0.561 | 0.442 | 0.423 | 0.468 |  | 0.741 | 0.627 | 0.583 | 0.601 |
|  |  | **SWITZERLAND** | |  |  |  |  |  |  |  |
| Low | 30+ | 0.078 | 0.151 | 0.161 | 0.124 |  | 0.079 | 0.129 | 0.151 | 0.147 |
|  | 25-30 | 0.473 | 0.530 | 0.530 | 0.499 |  | 0.274 | 0.395 | 0.409 | 0.358 |
|  | -25 | 0.449 | 0.319 | 0.309 | 0.378 |  | 0.646 | 0.476 | 0.440 | 0.496 |
| Middle | 30+ | 0.071 | 0.120 | 0.134 | 0.120 |  | 0.054 | 0.090 | 0.098 | 0.085 |
|  | 25-30 | 0.384 | 0.462 | 0.480 | 0.463 |  | 0.158 | 0.238 | 0.302 | 0.340 |
|  | -25 | 0.545 | 0.418 | 0.385 | 0.418 |  | 0.789 | 0.673 | 0.600 | 0.575 |
| High | 30+ | 0.037 | 0.084 | 0.084 | 0.054 |  | 0.031 | 0.041 | 0.041 | 0.037 |
|  | 25-30 | 0.336 | 0.424 | 0.451 | 0.437 |  | 0.091 | 0.195 | 0.239 | 0.217 |
|  | -25 | 0.628 | 0.493 | 0.466 | 0.509 |  | 0.877 | 0.764 | 0.720 | 0.746 |
|  |  | **TURIN AND TUSCANY (ITALIAN DATA APPLIED)** | | | | | | |  |  |
| Low | 30+ | 0.096 | 0.164 | 0.164 | 0.124 |  | 0.073 | 0.144 | 0.166 | 0.143 |
|  | 25-30 | 0.443 | 0.504 | 0.507 | 0.478 |  | 0.218 | 0.361 | 0.411 | 0.388 |
|  | -25 | 0.461 | 0.333 | 0.329 | 0.398 |  | 0.709 | 0.495 | 0.424 | 0.469 |
| Middle | 30+ | 0.070 | 0.112 | 0.107 | 0.077 |  | 0.034 | 0.068 | 0.083 | 0.077 |
|  | 25-30 | 0.399 | 0.507 | 0.522 | 0.481 |  | 0.152 | 0.235 | 0.297 | 0.329 |
|  | -25 | 0.531 | 0.381 | 0.372 | 0.441 |  | 0.814 | 0.697 | 0.619 | 0.594 |
| High | 30+ | 0.047 | 0.074 | 0.078 | 0.065 |  | 0.017 | 0.044 | 0.070 | 0.081 |
|  | 25-30 | 0.334 | 0.470 | 0.504 | 0.473 |  | 0.096 | 0.220 | 0.265 | 0.230 |
|  | -25 | 0.619 | 0.456 | 0.418 | 0.461 |  | 0.887 | 0.736 | 0.664 | 0.690 |

Note: extrapolated data marked “*”. BMI calculated on the basis of self-reported height and weight.

**Table S7: Prevalences of physical inactivity**

|  |  | Men | | | |  | Women | | | |
| --- | --- | --- | --- | --- | --- | --- | --- | --- | --- | --- |
|  | age | 30-44 | 45-59 | 60-69 | 70-79 |  | 30-44 | 45-59 | 60-69 | 70-79 |
| Education | Physical activity | **BARCELONA. BASQUE COUNTRY AND MADRID (SPANISH DATA APPLIED)** | | | | | | | |  |
| Low | Sedentary | 0.869 | 0.937 | 0.966 | 0.978 |  | 0.930 | 0.937 | 0.964 | 0.983 |
|  | Active | 0.131 | 0.063 | 0.034 | 0.022 |  | 0.070 | 0.063 | 0.036 | 0.017 |
| Middle | Sedentary | 0.762 | 0.863 | 0.913 | 0.937 |  | 0.861 | 0.866 | 0.843 | 0.802 |
|  | Active | 0.238 | 0.137 | 0.087 | 0.063 |  | 0.139 | 0.134 | 0.157 | 0.198 |
| High | Sedentary | 0.704 | 0.769 | 0.845 | 0.900 |  | 0.816 | 0.804 | 0.881 | 0.946 |
|  | Active | 0.296 | 0.232 | 0.156 | 0.100 |  | 0.184 | 0.196 | 0.119 | 0.054 |
|  |  | **BELGIUM** | | |  |  |  |  |  |  |
| Low | Sedentary | 0.392 | 0.354 | 0.399 | 0.488 |  | 0.504 | 0.410 | 0.484 | 0.636 |
|  | Active | 0.608 | 0.646 | 0.601 | 0.512 |  | 0.496 | 0.590 | 0.517 | 0.364 |
| Middle | Sedentary | 0.296 | 0.275 | 0.299 | 0.346 |  | 0.346 | 0.321 | 0.383 | 0.490 |
|  | Active | 0.704 | 0.725 | 0.701 | 0.654 |  | 0.654 | 0.679 | 0.617 | 0.510 |
| High | Sedentary | 0.188 | 0.211 | 0.296 | 0.423 |  | 0.276 | 0.241 | 0.322 | 0.479 |
|  | Active | 0.812 | 0.789 | 0.704 | 0.578 |  | 0.724 | 0.759 | 0.678 | 0.521 |
|  |  | **CZECH REPUBLIC** | |  |  |  |  |  |  |  |
| Low | Sedentary | 0.855 | 0.820 | 0.829 | 0.859 |  | 0.917 | 0.814 | 0.882 | 0.964 |
|  | Active | 0.146 | 0.180 | 0.171 | 0.141 |  | 0.083 | 0.187 | 0.118 | 0.036 |
| Middle | Sedentary | 0.848 | 0.808 | 0.831 | 0.876 |  | 0.897 | 0.912 | 0.932 | 0.949 |
|  | Active | 0.152 | 0.192 | 0.170 | 0.124 |  | 0.104 | 0.088 | 0.068 | 0.051 |
| High | Sedentary | 0.921 | 0.858 | 0.847 | 0.874 |  | 0.921 | 0.872 | 0.859 | 0.875 |
|  | Active | 0.079 | 0.142 | 0.153 | 0.126 |  | 0.079 | 0.128 | 0.141 | 0.125 |
|  |  | **DENMARK** | |  |  |  |  |  |  |  |
| Low | Sedentary | 0.221 | 0.209 | 0.239 | 0.293 |  | 0.232 | 0.157 | 0.209 | 0.362 |
|  | Active | 0.779 | 0.791 | 0.761 | 0.707 |  | 0.768 | 0.843 | 0.791 | 0.638 |
| Middle | Sedentary | 0.152 | 0.132 | 0.160 | 0.221 |  | 0.184 | 0.115 | 0.167 | 0.333 |
|  | Active | 0.848 | 0.868 | 0.840 | 0.779 |  | 0.816 | 0.885 | 0.833 | 0.667 |
| High | Sedentary | 0.110 | 0.116 | 0.149 | 0.203 |  | 0.093 | 0.084 | 0.133 | 0.255 |
|  | Active | 0.890 | 0.885 | 0.851 | 0.797 |  | 0.907 | 0.916 | 0.867 | 0.745 |
|  |  | **ESTONIA** | |  |  |  |  |  |  |  |
| Low | Sedentary | 0.775 | 0.836 | 0.828 | 0.783* |  | 0.732 | 0.782 | 0.720 | 0.582* |
|  | Active | 0.225 | 0.164 | 0.172 | 0.217* |  | 0.269 | 0.218 | 0.280 | 0.418* |
| Middle | Sedentary | 0.740 | 0.790 | 0.667 | 0.416* |  | 0.735 | 0.737 | 0.723 | 0.700* |
|  | Active | 0.260 | 0.211 | 0.333 | 0.584* |  | 0.266 | 0.263 | 0.277 | 0.300* |
| High | Sedentary | 0.596 | 0.725 | 0.893 | 0.968* |  | 0.679 | 0.703 | 0.635 | 0.515* |
|  | Active | 0.404 | 0.275 | 0.107 | 0.032* |  | 0.321 | 0.298 | 0.365 | 0.485* |
|  |  | **FINLAND** | | |  |  |  |  |  |  |
| Low | Sedentary | 0.499 | 0.440 | 0.301 | 0.175* |  | 0.408 | 0.378 | 0.288 | 0.195* |
|  | Active | 0.501 | 0.561 | 0.699 | 0.825* |  | 0.593 | 0.622 | 0.713 | 0.805* |
| Middle | Sedentary | 0.459 | 0.388 | 0.254 | 0.144* |  | 0.399 | 0.362 | 0.286 | 0.212* |
|  | Active | 0.541 | 0.612 | 0.746 | 0.856* |  | 0.601 | 0.638 | 0.714 | 0.788* |
| High | Sedentary | 0.415 | 0.345 | 0.306 | 0.287* |  | 0.401 | 0.360 | 0.306 | 0.255* |
|  | Active | 0.585 | 0.655 | 0.694 | 0.713* |  | 0.599 | 0.640 | 0.694 | 0.745* |
|  |  | **LITHUANIA** | |  |  |  |  |  |  |  |
| Low | Sedentary | 0.601 | 0.609 | 0.613 | 0.614 |  | 0.654 | 0.630 | 0.634 | 0.653* |
|  | Active | 0.399 | 0.391 | 0.387 | 0.386 |  | 0.346 | 0.370 | 0.366 | 0.347 |
| Middle | Sedentary | 0.598 | 0.575 | 0.455 | 0.308 |  | 0.615 | 0.535 | 0.375 | 0.224* |
|  | Active | 0.402 | 0.425 | 0.546 | 0.692 |  | 0.385 | 0.465 | 0.625 | 0.776 |
| High | Sedentary | 0.589 | 0.497 | 0.433 | 0.396 |  | 0.651 | 0.635 | 0.453 | 0.234* |
|  | Active | 0.411 | 0.503 | 0.567 | 0.604 |  | 0.349 | 0.365 | 0.547 | 0.766* |
|  |  | **NETHERLANDS** | |  |  |  |  |  |  |  |
| Low | Sedentary | 0.728 | 0.654 | 0.608 | 0.588 |  | 0.651 | 0.597 | 0.616 | 0.670 |
|  | Active | 0.272 | 0.346 | 0.392 | 0.412 |  | 0.350 | 0.403 | 0.384 | 0.330 |
| Middle | Sedentary | 0.761 | 0.689 | 0.630 | 0.588 |  | 0.644 | 0.595 | 0.605 | 0.646 |
|  | Active | 0.239 | 0.311 | 0.371 | 0.412 |  | 0.356 | 0.405 | 0.395 | 0.354 |
| High | Sedentary | 0.827 | 0.724 | 0.605 | 0.500 |  | 0.695 | 0.610 | 0.552 | 0.521 |
|  | Active | 0.173 | 0.276 | 0.395 | 0.500 |  | 0.305 | 0.391 | 0.448 | 0.479 |
|  |  | **NORWAY** | |  |  |  |  |  |  |  |
| Low | Sedentary | 0.578 | 0.684 | 0.776 | 0.839 |  | 0.885 | 0.791 | 0.862 | 0.949 |
|  | Active | 0.422 | 0.316 | 0.224 | 0.161 |  | 0.115 | 0.209 | 0.138 | 0.051 |
| Middle | Sedentary | 0.653 | 0.677 | 0.723 | 0.769 |  | 0.743 | 0.769 | 0.841 | 0.903 |
|  | Active | 0.347 | 0.323 | 0.277 | 0.231 |  | 0.257 | 0.231 | 0.159 | 0.097 |
| High | Sedentary | 0.591 | 0.655 | 0.676 | 0.671 |  | 0.702 | 0.752 | 0.822 | 0.878 |
|  | Active | 0.409 | 0.345 | 0.324 | 0.329 |  | 0.298 | 0.248 | 0.179 | 0.122 |
|  |  | **SWITZERLAND** | |  |  |  |  |  |  |  |
| Low | Sedentary | 0.304 | 0.260 | 0.276 | 0.326 |  | 0.290 | 0.268 | 0.307 | 0.381 |
|  | Active | 0.696 | 0.740 | 0.724 | 0.675 |  | 0.710 | 0.733 | 0.693 | 0.619 |
| Middle | Sedentary | 0.114 | 0.127 | 0.172 | 0.241 |  | 0.151 | 0.136 | 0.175 | 0.254 |
|  | Active | 0.886 | 0.873 | 0.828 | 0.759 |  | 0.849 | 0.864 | 0.825 | 0.746 |
| High | Sedentary | 0.091 | 0.107 | 0.137 | 0.176 |  | 0.115 | 0.143 | 0.192 | 0.255 |
|  | Active | 0.909 | 0.893 | 0.863 | 0.824 |  | 0.885 | 0.858 | 0.808 | 0.745 |
|  |  | **TURIN AND TUSCANY (ITALIAN DATA APPLIED)** | | | | | | |  |  |
| Low | Sedentary | 0.719 | 0.724 | 0.759 | 0.801 |  | 0.812 | 0.806 | 0.856 | 0.910 |
|  | Active | 0.281 | 0.276 | 0.241 | 0.199 |  | 0.188 | 0.194 | 0.144 | 0.090 |
| Middle | Sedentary | 0.593 | 0.670 | 0.720 | 0.748 |  | 0.728 | 0.756 | 0.813 | 0.866 |
|  | Active | 0.407 | 0.330 | 0.281 | 0.252 |  | 0.272 | 0.244 | 0.187 | 0.134 |
| High | Sedentary | 0.548 | 0.648 | 0.722 | 0.770 |  | 0.665 | 0.737 | 0.816 | 0.875 |
|  | Active | 0.452 | 0.352 | 0.278 | 0.230 |  | 0.335 | 0.263 | 0.184 | 0.125 |

Note: extrapolated data marked “*”. Self-reported leisure-time physical activity classified as ‘sedentary’ (less than once a week) or ‘active’ (once a week or more).

**Table S8: Prevalences of social participation**

|  |  | Men | | | |  | Women | | | |
| --- | --- | --- | --- | --- | --- | --- | --- | --- | --- | --- |
|  | **age** | 30-44 | 45-59 | 60-69 | 70-79 |  | 30-44 | 45-59 | 60-69 | 70-79 |
| **Education** | **Social participation** | **AUSTRIA** | |  |  |  |  |  |  |  |
| Low | No participation | 0.608 | 0.666 | 0.699 | 0.715 |  | 0.724 | 0.708 | 0.748 | 0.805 |
|  | Participation | 0.392 | 0.334 | 0.301 | 0.286 |  | 0.277 | 0.292 | 0.252 | 0.195 |
| Middle | No participation | 0.566 | 0.513 | 0.526 | 0.572 |  | 0.560 | 0.614 | 0.641 | 0.649 |
|  | Participation | 0.434 | 0.487 | 0.474 | 0.428 |  | 0.440 | 0.386 | 0.360 | 0.352 |
| High | No participation | 0.431 | 0.443 | 0.540 | 0.664 |  | 0.514 | 0.620 | 0.457 | 0.209 |
|  | Participation | 0.570 | 0.557 | 0.460 | 0.337 |  | 0.486 | 0.380 | 0.543 | 0.791 |
|  |  | **BARCELONA** | |  |  |  |  |  |  |  |
| Low | No participation | 0.758 | 0.778 | 0.789 | 0.794 |  | 0.865 | 0.799 | 0.782 | 0.801 |
|  | Participation | 0.242 | 0.222 | 0.211 | 0.206 |  | 0.135 | 0.201 | 0.218 | 0.199 |
| Middle | No participation | 0.693 | 0.658 | 0.731 | 0.829 |  | 0.849 | 0.694 | 0.872 | 0.981 |
|  | Participation | 0.307 | 0.343 | 0.269 | 0.171 |  | 0.151 | 0.306 | 0.128 | 0.019 |
| High | No participation | 0.624 | 0.523 | 0.495 | 0.513 |  | 0.621 | 0.535 | 0.673 | 0.850 |
|  | Participation | 0.376 | 0.477 | 0.505 | 0.487 |  | 0.379 | 0.465 | 0.327 | 0.150 |
|  |  | **BASQUE COUNTRY. MADRID AND BARCELONA (SPANISH DATA APPLIED)** | | | | | | | |  |
| Low | No participation | 0.758 | 0.778 | 0.789 | 0.794 |  | 0.865 | 0.799 | 0.782 | 0.801 |
|  | Participation | 0.242 | 0.222 | 0.211 | 0.206 |  | 0.135 | 0.201 | 0.218 | 0.199 |
| Middle | No participation | 0.693 | 0.658 | 0.731 | 0.829 |  | 0.849 | 0.694 | 0.872 | 0.981 |
|  | Participation | 0.307 | 0.343 | 0.269 | 0.171 |  | 0.151 | 0.306 | 0.128 | 0.019 |
| High | No participation | 0.624 | 0.523 | 0.495 | 0.513 |  | 0.621 | 0.535 | 0.673 | 0.850 |
|  | Participation | 0.376 | 0.477 | 0.505 | 0.487 |  | 0.379 | 0.465 | 0.327 | 0.150 |
|  |  | **BELGIUM** | |  |  |  |  |  |  |  |
| Low | No participation | 0.607 | 0.500 | 0.526 | 0.620 |  | 0.575 | 0.592 | 0.614 | 0.634 |
|  | Participation | 0.393 | 0.500 | 0.474 | 0.380 |  | 0.425 | 0.408 | 0.386 | 0.366 |
| Middle | No participation | 0.435 | 0.397 | 0.413 | 0.457 |  | 0.493 | 0.558 | 0.534 | 0.464 |
|  | Participation | 0.565 | 0.603 | 0.587 | 0.543 |  | 0.507 | 0.442 | 0.466 | 0.536 |
| High | No participation | 0.359 | 0.375 | 0.372 | 0.360 |  | 0.368 | 0.469 | 0.545 | 0.593 |
|  | Participation | 0.641 | 0.625 | 0.628 | 0.640 |  | 0.633 | 0.531 | 0.455 | 0.407 |
|  |  | **DENMARK** | |  |  |  |  |  |  |  |
| Low | No participation | 0.692 | 0.628 | 0.620 | 0.646 |  | 0.658 | 0.570 | 0.530 | 0.523 |
|  | Participation | 0.308 | 0.373 | 0.380 | 0.354 |  | 0.342 | 0.430 | 0.470 | 0.477 |
| Middle | No participation | 0.448 | 0.480 | 0.516 | 0.548 |  | 0.475 | 0.407 | 0.422 | 0.483 |
|  | Participation | 0.552 | 0.520 | 0.485 | 0.452 |  | 0.525 | 0.594 | 0.578 | 0.517 |
| High | No participation | 0.411 | 0.340 | 0.364 | 0.440 |  | 0.435 | 0.323 | 0.331 | 0.404 |
|  | Participation | 0.589 | 0.661 | 0.636 | 0.560 |  | 0.565 | 0.677 | 0.669 | 0.596 |
|  |  | **ENGLAND & WALES** | |  |  |  |  |  |  |  |
| Low | No participation | 0.515 | 0.512 | 0.563 | 0.633 |  | 0.738 | 0.578 | 0.534 | 0.570 |
|  | Participation | 0.485 | 0.488 | 0.437 | 0.367 |  | 0.263 | 0.423 | 0.466 | 0.430 |
| Middle | No participation | 0.407 | 0.224 | 0.168 | 0.165 |  | 0.420 | 0.261 | 0.239 | 0.283 |
|  | Participation | 0.593 | 0.776 | 0.832 | 0.835 |  | 0.580 | 0.739 | 0.761 | 0.717 |
| High | No participation | 0.354 | 0.376 | 0.378 | 0.367 |  | 0.342 | 0.290 | 0.327 | 0.414 |
|  | Participation | 0.646 | 0.624 | 0.622 | 0.633 |  | 0.658 | 0.710 | 0.673 | 0.586 |
|  |  | **FINLAND** | |  |  |  |  |  |  |  |
| Low | No participation | 0.724 | 0.638 | 0.611 | 0.626 |  | 0.825 | 0.649 | 0.583 | 0.612 |
|  | Participation | 0.276 | 0.362 | 0.389 | 0.375 |  | 0.175 | 0.351 | 0.417 | 0.388 |
| Middle | No participation | 0.552 | 0.609 | 0.578 | 0.501 |  | 0.553 | 0.508 | 0.580 | 0.696 |
|  | Participation | 0.448 | 0.391 | 0.422 | 0.499 |  | 0.447 | 0.492 | 0.420 | 0.305 |
| High | No participation | 0.493 | 0.498 | 0.684 | 0.865 |  | 0.437 | 0.380 | 0.414 | 0.497 |
|  | Participation | 0.507 | 0.502 | 0.316 | 0.136 |  | 0.563 | 0.621 | 0.586 | 0.503 |
|  |  | **FRANCE** | |  |  |  |  |  |  |  |
| Low | No participation | 0.771 | 0.592 | 0.577 | 0.663 |  | 0.744 | 0.683 | 0.677 | 0.704 |
|  | Participation | 0.229 | 0.408 | 0.424 | 0.337 |  | 0.256 | 0.317 | 0.323 | 0.296 |
| Middle | No participation | 0.563 | 0.574 | 0.600 | 0.630 |  | 0.584 | 0.528 | 0.508 | 0.512 |
|  | Participation | 0.437 | 0.426 | 0.400 | 0.370 |  | 0.416 | 0.472 | 0.492 | 0.488 |
| High | No participation | 0.409 | 0.349 | 0.376 | 0.450 |  | 0.366 | 0.257 | 0.323 | 0.498 |
|  | Participation | 0.591 | 0.651 | 0.624 | 0.551 |  | 0.635 | 0.743 | 0.677 | 0.502 |
|  |  | **HUNGARY** |  |  |  |  |  |  |  |  |
| Low | No participation | 0.847 | 0.864 | 0.889 | 0.912 |  | 0.881 | 0.833 | 0.839 | 0.872 |
|  | Participation | 0.153 | 0.136 | 0.111 | 0.088 |  | 0.119 | 0.167 | 0.161 | 0.128 |
| Middle | No participation | 0.786 | 0.737 | 0.722 | 0.731 |  | 0.691 | 0.779 | 0.888 | 0.950 |
|  | Participation | 0.214 | 0.263 | 0.278 | 0.269 |  | 0.309 | 0.221 | 0.113 | 0.050 |
| High | No participation | 0.454 | 0.637 | 0.652 | 0.577 |  | 0.599 | 0.629 | 0.744 | 0.851 |
|  | Participation | 0.546 | 0.363 | 0.348 | 0.423 |  | 0.401 | 0.371 | 0.256 | 0.149 |
|  |  | **NETHERLANDS** | |  |  |  |  |  |  |  |
| Low | No participation | 0.608 | 0.511 | 0.517 | 0.578 |  | 0.557 | 0.535 | 0.573 | 0.636 |
|  | Participation | 0.392 | 0.489 | 0.484 | 0.423 |  | 0.443 | 0.465 | 0.427 | 0.364 |
| Middle | No participation | 0.568 | 0.533 | 0.512 | 0.501 |  | 0.520 | 0.413 | 0.428 | 0.510 |
|  | Participation | 0.433 | 0.467 | 0.488 | 0.499 |  | 0.480 | 0.587 | 0.572 | 0.491 |
| High | No participation | 0.450 | 0.336 | 0.385 | 0.524 |  | 0.416 | 0.374 | 0.408 | 0.481 |
|  | Participation | 0.550 | 0.664 | 0.615 | 0.476 |  | 0.585 | 0.626 | 0.592 | 0.519 |
|  |  | **NORWAY** | |  |  |  |  |  |  |  |
| Low | No participation | 0.595 | 0.613 | 0.641 | 0.669 |  | 0.539 | 0.564 | 0.611 | 0.661 |
|  | Participation | 0.406 | 0.387 | 0.359 | 0.332 |  | 0.461 | 0.436 | 0.389 | 0.339 |
| Middle | No participation | 0.470 | 0.444 | 0.518 | 0.634 |  | 0.443 | 0.471 | 0.505 | 0.539 |
|  | Participation | 0.530 | 0.557 | 0.482 | 0.366 |  | 0.557 | 0.529 | 0.495 | 0.461 |
| High | No participation | 0.398 | 0.377 | 0.458 | 0.585 |  | 0.344 | 0.419 | 0.399 | 0.332 |
|  | Participation | 0.602 | 0.623 | 0.542 | 0.415 |  | 0.656 | 0.581 | 0.601 | 0.668 |
|  |  | **POLAND** | |  |  |  |  |  |  |  |
| Low | No participation | 0.928 | 0.932 | 0.969 | 0.990 |  | 0.996 | 0.973 | 0.931 | 0.907 |
|  | Participation | 0.072 | 0.068 | 0.031 | 0.010 |  | 0.004 | 0.027 | 0.069 | 0.093 |
| Middle | No participation | 0.883 | 0.897 | 0.878 | 0.837 |  | 0.945 | 0.912 | 0.876 | 0.846 |
|  | Participation | 0.118 | 0.104 | 0.122 | 0.163 |  | 0.055 | 0.088 | 0.124 | 0.154 |
| High | No participation | 0.669 | 0.664 | 0.672 | 0.686 |  | 0.666 | 0.738 | 0.836 | 0.906 |
|  | Participation | 0.331 | 0.336 | 0.328 | 0.314 |  | 0.334 | 0.262 | 0.164 | 0.094 |
|  |  | **SWEDEN** | |  |  |  |  |  |  |  |
| Low | No participation | 0.425 | 0.500 | 0.550 | 0.579 |  | 0.633 | 0.581 | 0.580 | 0.608 |
|  | Participation | 0.575 | 0.501 | 0.450 | 0.421 |  | 0.368 | 0.419 | 0.420 | 0.393 |
| Middle | No participation | 0.372 | 0.377 | 0.420 | 0.477 |  | 0.574 | 0.505 | 0.543 | 0.632 |
|  | Participation | 0.629 | 0.623 | 0.580 | 0.523 |  | 0.426 | 0.495 | 0.457 | 0.368 |
| High | No participation | 0.331 | 0.295 | 0.399 | 0.582 |  | 0.364 | 0.408 | 0.394 | 0.351 |
|  | Participation | 0.669 | 0.705 | 0.601 | 0.418 |  | 0.636 | 0.592 | 0.606 | 0.649 |
|  |  | **TURIN AND TUSCANY (ITALIAN DATA APPLIED)** | | | | | | |  |  |
| Low | No participation | 0.777 | 0.756 | 0.755 | 0.766 |  | 0.805 | 0.868 | 0.885 | 0.881 |
|  | Participation | 0.223 | 0.244 | 0.245 | 0.234 |  | 0.195 | 0.132 | 0.115 | 0.119 |
| Middle | No participation | 0.705 | 0.583 | 0.676 | 0.831 |  | 0.775 | 0.712 | 0.692 | 0.702 |
|  | Participation | 0.295 | 0.417 | 0.324 | 0.169 |  | 0.225 | 0.289 | 0.308 | 0.298 |
| High | No participation | 0.706 | 0.502 | 0.520 | 0.660 |  | 0.623 | 0.648 | 0.836 | 0.952 |
|  | Participation | 0.294 | 0.498 | 0.480 | 0.340 |  | 0.377 | 0.352 | 0.164 | 0.048 |

Self-reported social participation classified as ‘no participation’ (membership of null clubs, associations, etc.) and ‘participation’ (membership of at least one club, association, etc.).

**Table S9: Prevalences of lower income quartiles**

|  |  | Men | | | |  | Women | | | |
| --- | --- | --- | --- | --- | --- | --- | --- | --- | --- | --- |
|  | age | 30-44 | 45-59 | 60-69 | 70-79 |  | 30-44 | 45-59 | 60-69 | 70-79 |
| Education | Income | **BELGIUM** | |  |  |  |  |  |  |  |
| Low | Lowest | 0.306 | 0.287 | 0.344 | 0.416 |  | 0.402 | 0.316 | 0.331 | 0.388 |
|  | Second | 0.264 | 0.307 | 0.334 | 0.346 |  | 0.284 | 0.336 | 0.361 | 0.365 |
|  | Third | 0.305 | 0.286 | 0.234 | 0.180 |  | 0.204 | 0.225 | 0.212 | 0.185 |
|  | Highest | 0.125 | 0.120 | 0.088 | 0.058 |  | 0.110 | 0.124 | 0.096 | 0.062 |
| Middle | Lowest | 0.202 | 0.154 | 0.173 | 0.212 |  | 0.242 | 0.210 | 0.207 | 0.220 |
|  | Second | 0.221 | 0.259 | 0.302 | 0.342 |  | 0.224 | 0.229 | 0.257 | 0.298 |
|  | Third | 0.347 | 0.268 | 0.251 | 0.267 |  | 0.326 | 0.249 | 0.238 | 0.261 |
|  | Highest | 0.230 | 0.319 | 0.274 | 0.179 |  | 0.208 | 0.312 | 0.298 | 0.222 |
| High | Lowest | 0.100 | 0.085 | 0.104 | 0.131 |  | 0.102 | 0.100 | 0.131 | 0.170 |
|  | Second | 0.103 | 0.107 | 0.137 | 0.189 |  | 0.133 | 0.141 | 0.159 | 0.181 |
|  | Third | 0.283 | 0.224 | 0.218 | 0.240 |  | 0.305 | 0.236 | 0.239 | 0.282 |
|  | Highest | 0.514 | 0.585 | 0.542 | 0.440 |  | 0.460 | 0.523 | 0.471 | 0.367 |
|  |  | **CZECH REPUBLIC** | |  |  |  |  |  |  |  |
| Low | Lowest | 0.470 | 0.481 | 0.333 | 0.086 |  | 0.636 | 0.319 | 0.160 | 0.128 |
|  | Second | 0.206 | 0.219 | 0.293 | 0.407 |  | 0.170 | 0.382 | 0.451 | 0.400 |
|  | Third | 0.189 | 0.233 | 0.293 | 0.356 |  | 0.150 | 0.153 | 0.230 | 0.375 |
|  | Highest | 0.135 | 0.067 | 0.081 | 0.152 |  | 0.044 | 0.146 | 0.159 | 0.096 |
| Middle | Lowest | 0.447 | 0.261 | 0.133 | 0.059 |  | 0.527 | 0.218 | 0.079 | 0.107 |
|  | Second | 0.136 | 0.238 | 0.373 | 0.503 |  | 0.203 | 0.358 | 0.372 | 0.295 |
|  | Third | 0.228 | 0.263 | 0.266 | 0.250 |  | 0.092 | 0.239 | 0.352 | 0.386 |
|  | Highest | 0.189 | 0.238 | 0.228 | 0.188 |  | 0.179 | 0.185 | 0.198 | 0.212 |
| High | Lowest | 0.282 | 0.161 | 0.057 | 0.000 |  | 0.244 | 0.152 | 0.080 | 0.000 |
|  | Second | 0.264 | 0.114 | 0.129 | 0.212 |  | 0.217 | 0.210 | 0.103 | 0.000 |
|  | Third | 0.087 | 0.156 | 0.234 | 0.302 |  | 0.112 | 0.218 | 0.381 | 0.539 |
|  | Highest | 0.367 | 0.568 | 0.581 | 0.486 |  | 0.427 | 0.421 | 0.436 | 0.461 |
|  |  | **DENMARK** | | |  |  |  |  |  |  |
| Low | Lowest | 0.258 | 0.223 | 0.607 | 0.811 |  | 0.355 | 0.301 | 0.659 | 0.846 |
|  | Second | 0.291 | 0.263 | 0.208 | 0.156 |  | 0.298 | 0.283 | 0.208 | 0.134 |
|  | Third | 0.325 | 0.313 | 0.118 | 0.024 |  | 0.256 | 0.230 | 0.075 | 0.014 |
|  | Highest | 0.127 | 0.202 | 0.067 | 0.009 |  | 0.092 | 0.186 | 0.058 | 0.006 |
| Middle | Lowest | 0.149 | 0.098 | 0.463 | 0.699 |  | 0.284 | 0.152 | 0.540 | 0.702 |
|  | Second | 0.282 | 0.253 | 0.232 | 0.218 |  | 0.259 | 0.236 | 0.231 | 0.236 |
|  | Third | 0.385 | 0.312 | 0.156 | 0.059 |  | 0.334 | 0.265 | 0.138 | 0.058 |
|  | Highest | 0.184 | 0.337 | 0.149 | 0.025 |  | 0.123 | 0.346 | 0.091 | 0.005 |
| High | Lowest | 0.094 | 0.076 | 0.200 | 0.340 |  | 0.164 | 0.053 | 0.355 | 0.549 |
|  | Second | 0.218 | 0.151 | 0.181 | 0.277 |  | 0.249 | 0.182 | 0.203 | 0.278 |
|  | Third | 0.378 | 0.253 | 0.198 | 0.180 |  | 0.361 | 0.280 | 0.197 | 0.137 |
|  | Highest | 0.310 | 0.520 | 0.421 | 0.204 |  | 0.226 | 0.485 | 0.245 | 0.037 |
|  |  | **ENGLAND & WALES** | |  |  |  |  |  |  |  |
| Low | Lowest | 0.338 | 0.263 | 0.407 | 0.556 |  | 0.453 | 0.339 | 0.454 | 0.612 |
|  | Second | 0.312 | 0.319 | 0.319 | 0.316 |  | 0.248 | 0.316 | 0.310 | 0.264 |
|  | Third | 0.241 | 0.252 | 0.168 | 0.087 |  | 0.193 | 0.221 | 0.159 | 0.089 |
|  | Highest | 0.110 | 0.166 | 0.106 | 0.042 |  | 0.106 | 0.124 | 0.078 | 0.036 |
| Middle | Lowest | 0.152 | 0.121 | 0.211 | 0.263 |  | 0.189 | 0.128 | 0.249 | 0.316 |
|  | Second | 0.254 | 0.216 | 0.273 | 0.395 |  | 0.275 | 0.221 | 0.280 | 0.413 |
|  | Third | 0.339 | 0.341 | 0.299 | 0.244 |  | 0.320 | 0.319 | 0.265 | 0.200 |
|  | Highest | 0.256 | 0.323 | 0.217 | 0.098 |  | 0.217 | 0.332 | 0.207 | 0.072 |
| High | Lowest | 0.065 | 0.031 | 0.094 | 0.145 |  | 0.097 | 0.022 | 0.150 | 0.223 |
|  | Second | 0.143 | 0.133 | 0.186 | 0.295 |  | 0.116 | 0.165 | 0.250 | 0.357 |
|  | Third | 0.277 | 0.314 | 0.328 | 0.327 |  | 0.287 | 0.298 | 0.313 | 0.328 |
|  | Highest | 0.514 | 0.521 | 0.392 | 0.234 |  | 0.500 | 0.515 | 0.288 | 0.092 |
|  |  | **FINLAND** | |  |  |  |  |  |  |  |
| Low | Lowest | 0.327 | 0.327 | 0.348 | 0.377 |  | 0.565 | 0.378 | 0.240 | 0.205 |
|  | Second | 0.337 | 0.358 | 0.325 | 0.271 |  | 0.284 | 0.290 | 0.306 | 0.327 |
|  | Third | 0.236 | 0.183 | 0.187 | 0.222 |  | 0.076 | 0.215 | 0.304 | 0.303 |
|  | Highest | 0.100 | 0.133 | 0.140 | 0.131 |  | 0.076 | 0.117 | 0.149 | 0.166 |
| Middle | Lowest | 0.312 | 0.241 | 0.258 | 0.265 |  | 0.323 | 0.262 | 0.221 | 0.196 |
|  | Second | 0.271 | 0.280 | 0.200 | 0.116 |  | 0.263 | 0.269 | 0.256 | 0.235 |
|  | Third | 0.260 | 0.286 | 0.264 | 0.223 |  | 0.178 | 0.220 | 0.255 | 0.279 |
|  | Highest | 0.158 | 0.193 | 0.279 | 0.397 |  | 0.237 | 0.249 | 0.269 | 0.291 |
| High | Lowest | 0.120 | 0.091 | 0.094 | 0.045 |  | 0.128 | 0.156 | 0.117 | 0.050 |
|  | Second | 0.178 | 0.160 | 0.063 | 0.017 |  | 0.248 | 0.198 | 0.183 | 0.187 |
|  | Third | 0.324 | 0.323 | 0.242 | 0.155 |  | 0.220 | 0.228 | 0.225 | 0.217 |
|  | Highest | 0.378 | 0.427 | 0.601 | 0.783 |  | 0.404 | 0.418 | 0.475 | 0.547 |
|  |  | **FRANCE** | | |  |  |  |  |  |  |
| Low | Lowest | 0.420 | 0.371 | 0.347 | 0.350 |  | 0.427 | 0.395 | 0.348 | 0.316 |
|  | Second | 0.308 | 0.261 | 0.241 | 0.235 |  | 0.374 | 0.302 | 0.286 | 0.300 |
|  | Third | 0.182 | 0.230 | 0.256 | 0.264 |  | 0.120 | 0.193 | 0.222 | 0.213 |
|  | Highest | 0.090 | 0.138 | 0.157 | 0.151 |  | 0.078 | 0.111 | 0.144 | 0.171 |
| Middle | Lowest | 0.335 | 0.221 | 0.235 | 0.311 |  | 0.207 | 0.236 | 0.200 | 0.149 |
|  | Second | 0.314 | 0.260 | 0.267 | 0.308 |  | 0.399 | 0.255 | 0.208 | 0.207 |
|  | Third | 0.163 | 0.328 | 0.303 | 0.183 |  | 0.231 | 0.306 | 0.332 | 0.322 |
|  | Highest | 0.188 | 0.191 | 0.195 | 0.198 |  | 0.163 | 0.204 | 0.261 | 0.322 |
| High | Lowest | 0.120 | 0.112 | 0.045 | 0.000 |  | 0.063 | 0.082 | 0.079 | 0.031 |
|  | Second | 0.149 | 0.167 | 0.110 | 0.000 |  | 0.228 | 0.177 | 0.090 | 0.038 |
|  | Third | 0.245 | 0.266 | 0.278 | 0.276 |  | 0.273 | 0.229 | 0.222 | 0.236 |
|  | Highest | 0.486 | 0.455 | 0.567 | 0.724 |  | 0.437 | 0.512 | 0.609 | 0.695 |
|  |  | **HUNGARY** | |  |  |  |  |  |  |  |
| Low | Lowest | 0.506 | 0.438 | 0.337 | 0.234 |  | 0.490 | 0.408 | 0.372 | 0.371 |
|  | Second | 0.249 | 0.283 | 0.337 | 0.396 |  | 0.276 | 0.293 | 0.318 | 0.343 |
|  | Third | 0.120 | 0.179 | 0.232 | 0.269 |  | 0.136 | 0.196 | 0.216 | 0.206 |
|  | Highest | 0.125 | 0.100 | 0.095 | 0.101 |  | 0.098 | 0.104 | 0.095 | 0.081 |
| Middle | Lowest | 0.225 | 0.223 | 0.203 | 0.177 |  | 0.202 | 0.193 | 0.208 | 0.199 |
|  | Second | 0.201 | 0.233 | 0.272 | 0.311 |  | 0.234 | 0.221 | 0.275 | 0.371 |
|  | Third | 0.269 | 0.288 | 0.288 | 0.278 |  | 0.281 | 0.273 | 0.268 | 0.265 |
|  | Highest | 0.305 | 0.256 | 0.237 | 0.235 |  | 0.283 | 0.313 | 0.250 | 0.165 |
| High | Lowest | 0.063 | 0.046 | 0.044 | 0.047 |  | 0.044 | 0.037 | 0.087 | 0.121 |
|  | Second | 0.144 | 0.088 | 0.093 | 0.132 |  | 0.126 | 0.113 | 0.167 | 0.288 |
|  | Third | 0.165 | 0.190 | 0.247 | 0.324 |  | 0.247 | 0.236 | 0.237 | 0.245 |
|  | Highest | 0.628 | 0.676 | 0.616 | 0.497 |  | 0.583 | 0.614 | 0.509 | 0.346 |
|  |  | **NETHERLANDS** | |  |  |  |  |  |  |  |
| Low | Lowest | 0.467 | 0.323 | 0.269 | 0.276 |  | 0.358 | 0.343 | 0.291 | 0.227 |
|  | Second | 0.235 | 0.382 | 0.414 | 0.367 |  | 0.311 | 0.257 | 0.268 | 0.313 |
|  | Third | 0.177 | 0.145 | 0.166 | 0.219 |  | 0.187 | 0.238 | 0.264 | 0.269 |
|  | Highest | 0.121 | 0.150 | 0.152 | 0.138 |  | 0.144 | 0.162 | 0.178 | 0.192 |
| Middle | Lowest | 0.285 | 0.207 | 0.188 | 0.199 |  | 0.239 | 0.219 | 0.185 | 0.134 |
|  | Second | 0.312 | 0.334 | 0.321 | 0.293 |  | 0.288 | 0.265 | 0.216 | 0.169 |
|  | Third | 0.216 | 0.263 | 0.264 | 0.237 |  | 0.269 | 0.287 | 0.325 | 0.371 |
|  | Highest | 0.188 | 0.196 | 0.227 | 0.272 |  | 0.204 | 0.229 | 0.274 | 0.326 |
| High | Lowest | 0.088 | 0.059 | 0.040 | 0.018 |  | 0.133 | 0.079 | 0.094 | 0.067 |
|  | Second | 0.225 | 0.196 | 0.145 | 0.101 |  | 0.145 | 0.192 | 0.098 | 0.029 |
|  | Third | 0.284 | 0.317 | 0.343 | 0.362 |  | 0.385 | 0.284 | 0.267 | 0.293 |
|  | Highest | 0.403 | 0.428 | 0.472 | 0.519 |  | 0.337 | 0.445 | 0.540 | 0.610 |
|  |  | **NORWAY** | |  |  |  |  |  |  |  |
| Low | Lowest | 0.286 | 0.188 | 0.456 | 0.697 |  | 0.316 | 0.242 | 0.551 | 0.770 |
|  | Second | 0.380 | 0.319 | 0.255 | 0.203 |  | 0.316 | 0.300 | 0.240 | 0.178 |
|  | Third | 0.251 | 0.272 | 0.167 | 0.073 |  | 0.183 | 0.284 | 0.128 | 0.026 |
|  | Highest | 0.084 | 0.221 | 0.123 | 0.027 |  | 0.185 | 0.174 | 0.081 | 0.026 |
| Middle | Lowest | 0.210 | 0.119 | 0.264 | 0.474 |  | 0.284 | 0.125 | 0.319 | 0.552 |
|  | Second | 0.334 | 0.248 | 0.213 | 0.206 |  | 0.332 | 0.250 | 0.227 | 0.235 |
|  | Third | 0.272 | 0.318 | 0.266 | 0.185 |  | 0.244 | 0.297 | 0.238 | 0.149 |
|  | Highest | 0.183 | 0.316 | 0.257 | 0.135 |  | 0.140 | 0.329 | 0.217 | 0.064 |
| High | Lowest | 0.070 | 0.040 | 0.092 | 0.144 |  | 0.108 | 0.051 | 0.149 | 0.288 |
|  | Second | 0.176 | 0.115 | 0.156 | 0.283 |  | 0.271 | 0.180 | 0.197 | 0.275 |
|  | Third | 0.349 | 0.278 | 0.243 | 0.230 |  | 0.330 | 0.294 | 0.242 | 0.195 |
|  | Highest | 0.405 | 0.567 | 0.509 | 0.343 |  | 0.291 | 0.475 | 0.412 | 0.242 |
|  |  | **POLAND** | |  |  |  |  |  |  |  |
| Low | Lowest | 0.416 | 0.349 | 0.300 | 0.272 |  | 0.451 | 0.358 | 0.316 | 0.295 |
|  | Second | 0.276 | 0.267 | 0.265 | 0.266 |  | 0.318 | 0.325 | 0.281 | 0.223 |
|  | Third | 0.212 | 0.249 | 0.273 | 0.287 |  | 0.148 | 0.213 | 0.260 | 0.283 |
|  | Highest | 0.096 | 0.135 | 0.163 | 0.176 |  | 0.083 | 0.104 | 0.144 | 0.200 |
| Middle | Lowest | 0.262 | 0.248 | 0.239 | 0.239 |  | 0.290 | 0.205 | 0.163 | 0.103 |
|  | Second | 0.253 | 0.217 | 0.206 | 0.209 |  | 0.274 | 0.317 | 0.240 | 0.142 |
|  | Third | 0.301 | 0.299 | 0.287 | 0.271 |  | 0.241 | 0.231 | 0.263 | 0.321 |
|  | Highest | 0.184 | 0.236 | 0.268 | 0.281 |  | 0.195 | 0.247 | 0.334 | 0.435 |
| High | Lowest | 0.042 | 0.040 | 0.009 | 0.054 |  | 0.071 | 0.084 | 0.155 | 0.058 |
|  | Second | 0.109 | 0.088 | 0.000 | 0.000 |  | 0.118 | 0.259 | 0.117 | 0.020 |
|  | Third | 0.317 | 0.168 | 0.157 | 0.208 |  | 0.233 | 0.229 | 0.283 | 0.374 |
|  | Highest | 0.531 | 0.705 | 0.752 | 0.738 |  | 0.578 | 0.429 | 0.445 | 0.548 |
|  |  | **SCOTLAND** | |  |  |  |  |  |  |  |
| Low | Lowest | 0.438 | 0.363 | 0.398 | 0.427 |  | 0.536 | 0.408 | 0.355 | 0.340 |
|  | Second | 0.238 | 0.277 | 0.346 | 0.428 |  | 0.229 | 0.338 | 0.440 | 0.517 |
|  | Third | 0.241 | 0.235 | 0.171 | 0.106 |  | 0.171 | 0.190 | 0.154 | 0.106 |
|  | Highest | 0.083 | 0.125 | 0.085 | 0.038 |  | 0.063 | 0.065 | 0.051 | 0.037 |
| Middle | Lowest | 0.178 | 0.172 | 0.260 | 0.321 |  | 0.235 | 0.168 | 0.218 | 0.256 |
|  | Second | 0.256 | 0.242 | 0.295 | 0.387 |  | 0.266 | 0.281 | 0.344 | 0.431 |
|  | Third | 0.299 | 0.335 | 0.285 | 0.209 |  | 0.282 | 0.296 | 0.274 | 0.238 |
|  | Highest | 0.267 | 0.252 | 0.161 | 0.083 |  | 0.218 | 0.255 | 0.165 | 0.075 |
| High | Lowest | 0.072 | 0.045 | 0.064 | 0.070 |  | 0.080 | 0.035 | 0.109 | 0.159 |
|  | Second | 0.106 | 0.107 | 0.147 | 0.222 |  | 0.115 | 0.143 | 0.185 | 0.232 |
|  | Third | 0.242 | 0.240 | 0.294 | 0.382 |  | 0.277 | 0.305 | 0.375 | 0.462 |
|  | Highest | 0.579 | 0.608 | 0.495 | 0.325 |  | 0.529 | 0.517 | 0.331 | 0.147 |
|  |  | **SWEDEN** | |  |  |  |  |  |  |  |
| Low | Lowest | 0.330 | 0.381 | 0.378 | 0.352 |  | 0.435 | 0.240 | 0.228 | 0.331 |
|  | Second | 0.293 | 0.227 | 0.223 | 0.252 |  | 0.255 | 0.322 | 0.307 | 0.251 |
|  | Third | 0.276 | 0.256 | 0.235 | 0.219 |  | 0.164 | 0.286 | 0.321 | 0.286 |
|  | Highest | 0.101 | 0.137 | 0.164 | 0.177 |  | 0.147 | 0.152 | 0.145 | 0.132 |
| Middle | Lowest | 0.251 | 0.256 | 0.228 | 0.143 |  | 0.259 | 0.245 | 0.222 | 0.197 |
|  | Second | 0.240 | 0.196 | 0.175 | 0.167 |  | 0.325 | 0.194 | 0.101 | 0.053 |
|  | Third | 0.291 | 0.313 | 0.249 | 0.168 |  | 0.208 | 0.250 | 0.282 | 0.302 |
|  | Highest | 0.218 | 0.235 | 0.348 | 0.522 |  | 0.208 | 0.311 | 0.395 | 0.448 |
| High | Lowest | 0.091 | 0.104 | 0.136 | 0.058 |  | 0.143 | 0.099 | 0.116 | 0.144 |
|  | Second | 0.165 | 0.153 | 0.036 | 0.004 |  | 0.233 | 0.239 | 0.177 | 0.110 |
|  | Third | 0.386 | 0.295 | 0.278 | 0.299 |  | 0.209 | 0.274 | 0.298 | 0.292 |
|  | Highest | 0.358 | 0.449 | 0.551 | 0.639 |  | 0.416 | 0.388 | 0.409 | 0.454 |
|  |  | **SWITZERLAND** | |  |  |  |  |  |  |  |
| Low | Lowest | 0.480 | 0.428 | 0.380 | 0.343 |  | 0.418 | 0.434 | 0.410 | 0.369 |
|  | Second | 0.225 | 0.321 | 0.344 | 0.317 |  | 0.283 | 0.270 | 0.276 | 0.293 |
|  | Third | 0.182 | 0.131 | 0.145 | 0.199 |  | 0.167 | 0.188 | 0.206 | 0.219 |
|  | Highest | 0.113 | 0.121 | 0.131 | 0.141 |  | 0.132 | 0.108 | 0.108 | 0.119 |
| Middle | Lowest | 0.282 | 0.215 | 0.230 | 0.285 |  | 0.280 | 0.220 | 0.199 | 0.184 |
|  | Second | 0.313 | 0.327 | 0.313 | 0.287 |  | 0.310 | 0.282 | 0.208 | 0.139 |
|  | Third | 0.219 | 0.257 | 0.241 | 0.201 |  | 0.219 | 0.280 | 0.352 | 0.418 |
|  | Highest | 0.186 | 0.201 | 0.216 | 0.228 |  | 0.191 | 0.218 | 0.241 | 0.259 |
| High | Lowest | 0.082 | 0.081 | 0.073 | 0.056 |  | 0.119 | 0.151 | 0.170 | 0.155 |
|  | Second | 0.227 | 0.181 | 0.136 | 0.102 |  | 0.159 | 0.165 | 0.162 | 0.156 |
|  | Third | 0.288 | 0.309 | 0.319 | 0.321 |  | 0.264 | 0.298 | 0.238 | 0.156 |
|  | Highest | 0.403 | 0.428 | 0.472 | 0.521 |  | 0.458 | 0.387 | 0.430 | 0.534 |

Self-reported net equivalent household income classified in quartiles based on national distributions.

**Table S10: Prevalences of economic inactivity**

|  |  | Men | | | |  | Women | | | |
| --- | --- | --- | --- | --- | --- | --- | --- | --- | --- | --- |
|  | age | 30-44 | 45-59 | 60-69 | 70-79 |  | 30-44 | 45-59 | 60-69 | 70-79 |
| Education | Activity status | **AUSTRIA** | |  |  |  |  |  |  |  |
| Low | Inactive | 0.085 | 0.242 | 0.957 | 1.000 |  | 0.301 | 0.533 | 0.978 | 1.000 |
|  | Active | 0.915 | 0.758 | 0.043 | 0.000 |  | 0.699 | 0.467 | 0.022 | 0.000 |
| Middle | Inactive | 0.029 | 0.166 | 0.932 | 0.999 |  | 0.201 | 0.427 | 0.964 | 1.000 |
|  | Active | 0.971 | 0.834 | 0.068 | 0.001 |  | 0.799 | 0.573 | 0.036 | 0.000 |
| High | Inactive | 0.021 | 0.100 | 0.757 | 0.993 |  | 0.115 | 0.233 | 0.877 | 0.997 |
|  | Active | 0.979 | 0.900 | 0.243 | 0.007 |  | 0.885 | 0.767 | 0.123 | 0.003 |
|  |  | **MADRID** | | | | | |  |  |  |
| Low | Inactive | 0.134 | 0.203 | 0.732 | 0.983 |  | 0.507 | 0.691 | 0.901 | 0.977 |
|  | Active | 0.866 | 0.797 | 0.268 | 0.017 |  | 0.493 | 0.309 | 0.099 | 0.023 |
| Middle | Inactive | 0.086 | 0.164 | 0.669 | 0.973 |  | 0.318 | 0.435 | 0.798 | 0.967 |
|  | Active | 0.914 | 0.836 | 0.331 | 0.027 |  | 0.682 | 0.565 | 0.202 | 0.033 |
| High | Inactive | 0.070 | 0.117 | 0.492 | 0.921 |  | 0.180 | 0.250 | 0.656 | 0.946 |
|  | Active | 0.930 | 0.884 | 0.508 | 0.079 |  | 0.820 | 0.750 | 0.344 | 0.054 |
|  |  | **BASQUE COUNTRY** | |  |  |  |  |  |  |  |
| Low | Inactive | 0.079 | 0.161 | 0.821 | 0.996 |  | 0.437 | 0.660 | 0.921 | 0.989 |
|  | Active | 0.921 | 0.839 | 0.179 | 0.004 |  | 0.563 | 0.341 | 0.079 | 0.011 |
| Middle | Inactive | 0.045 | 0.107 | 0.723 | 0.992 |  | 0.255 | 0.382 | 0.803 | 0.976 |
|  | Active | 0.955 | 0.894 | 0.277 | 0.008 |  | 0.745 | 0.618 | 0.197 | 0.024 |
| High | Inactive | 0.057 | 0.086 | 0.574 | 0.978 |  | 0.132 | 0.205 | 0.643 | 0.954 |
|  | Active | 0.943 | 0.914 | 0.427 | 0.022 |  | 0.868 | 0.795 | 0.357 | 0.046 |
|  |  | **DENMARK** | |  |  |  |  |  |  |  |
| Low | Inactive | 0.209 | 0.251 | 0.670 | 0.957 |  | 0.285 | 0.349 | 0.846 | 0.992 |
|  | Active | 0.791 | 0.749 | 0.330 | 0.043 |  | 0.715 | 0.651 | 0.154 | 0.008 |
| Middle | Inactive | 0.049 | 0.115 | 0.619 | 0.971 |  | 0.082 | 0.160 | 0.758 | 0.991 |
|  | Active | 0.951 | 0.885 | 0.381 | 0.029 |  | 0.918 | 0.840 | 0.242 | 0.009 |
| High | Inactive | 0.067 | 0.076 | 0.427 | 0.939 |  | 0.102 | 0.096 | 0.623 | 0.988 |
|  | Active | 0.933 | 0.924 | 0.573 | 0.061 |  | 0.898 | 0.904 | 0.377 | 0.012 |
|  |  | **ENGLAND & WALES** | |  |  |  |  |  |  |  |
| Low | Inactive | 0.129 | 0.236 | 0.683 | 0.957 |  | 0.347 | 0.401 | 0.832 | 0.986 |
|  | Active | 0.871 | 0.765 | 0.317 | 0.043 |  | 0.653 | 0.599 | 0.168 | 0.014 |
| Middle | Inactive | 0.063 | 0.147 | 0.581 | 0.939 |  | 0.215 | 0.253 | 0.747 | 0.982 |
|  | Active | 0.937 | 0.853 | 0.419 | 0.061 |  | 0.785 | 0.747 | 0.253 | 0.018 |
| High | Inactive | 0.043 | 0.124 | 0.569 | 0.941 |  | 0.161 | 0.211 | 0.748 | 0.986 |
|  | Active | 0.957 | 0.876 | 0.431 | 0.059 |  | 0.839 | 0.789 | 0.252 | 0.014 |
|  |  | **FINLAND** | | |  |  |  |  |  |  |
| Low | Inactive | 0.169 | 0.186 | 0.676 | 0.978 |  | 0.260 | 0.191 | 0.677 | 0.982 |
|  | Active | 0.831 | 0.814 | 0.324 | 0.022 |  | 0.740 | 0.809 | 0.323 | 0.018 |
| Middle | Inactive | 0.079 | 0.124 | 0.559 | 0.954 |  | 0.173 | 0.121 | 0.565 | 0.974 |
|  | Active | 0.922 | 0.876 | 0.441 | 0.046 |  | 0.827 | 0.879 | 0.435 | 0.026 |
| High | Inactive | 0.058 | 0.074 | 0.473 | 0.959 |  | 0.126 | 0.074 | 0.466 | 0.971 |
|  | Active | 0.942 | 0.927 | 0.527 | 0.041 |  | 0.874 | 0.926 | 0.534 | 0.029 |
|  |  | **HUNGARY** | |  |  |  |  |  |  |  |
| Low | Inactive | 0.191 | 0.418 | 0.945 | 0.999 |  | 0.360 | 0.586 | 0.973 | 1.000 |
|  | Active | 0.809 | 0.582 | 0.055 | 0.001 |  | 0.640 | 0.414 | 0.027 | 0.000 |
| Middle | Inactive | 0.077 | 0.259 | 0.861 | 0.994 |  | 0.202 | 0.360 | 0.906 | 0.997 |
|  | Active | 0.923 | 0.742 | 0.139 | 0.006 |  | 0.798 | 0.641 | 0.094 | 0.003 |
| High | Inactive | 0.022 | 0.162 | 0.676 | 0.947 |  | 0.154 | 0.210 | 0.752 | 0.987 |
|  | Active | 0.978 | 0.839 | 0.324 | 0.053 |  | 0.846 | 0.790 | 0.248 | 0.013 |
|  |  | **NORWAY** | |  |  |  |  |  |  |  |
| Low | Inactive | 0.224 | 0.266 | 0.666 | 0.950 |  | 0.404 | 0.421 | 0.826 | 0.984 |
|  | Active | 0.776 | 0.734 | 0.334 | 0.050 |  | 0.596 | 0.579 | 0.174 | 0.016 |
| Middle | Inactive | 0.127 | 0.148 | 0.546 | 0.942 |  | 0.247 | 0.226 | 0.667 | 0.969 |
|  | Active | 0.873 | 0.852 | 0.454 | 0.058 |  | 0.753 | 0.774 | 0.334 | 0.031 |
| High | Inactive | 0.070 | 0.072 | 0.384 | 0.918 |  | 0.133 | 0.106 | 0.494 | 0.956 |
|  | Active | 0.930 | 0.928 | 0.616 | 0.082 |  | 0.867 | 0.894 | 0.506 | 0.044 |
|  |  | **SCOTLAND** | |  |  |  |  |  |  |  |
| Low | Inactive | 0.254 | 0.356 | 0.763 | 0.967 |  | 0.531 | 0.450 | 0.902 | 0.997 |
|  | Active | 0.746 | 0.644 | 0.237 | 0.033 |  | 0.469 | 0.551 | 0.098 | 0.003 |
| Middle | Inactive | 0.111 | 0.176 | 0.643 | 0.964 |  | 0.253 | 0.292 | 0.824 | 0.992 |
|  | Active | 0.889 | 0.824 | 0.357 | 0.036 |  | 0.747 | 0.708 | 0.176 | 0.008 |
| High | Inactive | 0.027 | 0.096 | 0.560 | 0.952 |  | 0.161 | 0.165 | 0.721 | 0.989 |
|  | Active | 0.973 | 0.904 | 0.440 | 0.048 |  | 0.839 | 0.835 | 0.279 | 0.011 |
|  |  | **SWEDEN** |  |  |  |  |  |  |  |  |
| Low | Inactive | 0.238 | 0.230 | 0.708 | 0.979 |  | 0.386 | 0.323 | 0.797 | 0.989 |
|  | Active | 0.762 | 0.770 | 0.292 | 0.021 |  | 0.614 | 0.678 | 0.203 | 0.011 |
| Middle | Inactive | 0.139 | 0.183 | 0.646 | 0.966 |  | 0.205 | 0.195 | 0.708 | 0.985 |
|  | Active | 0.862 | 0.817 | 0.354 | 0.034 |  | 0.795 | 0.806 | 0.292 | 0.015 |
| High | Inactive | 0.112 | 0.122 | 0.513 | 0.945 |  | 0.140 | 0.098 | 0.567 | 0.981 |
|  | Active | 0.888 | 0.878 | 0.487 | 0.055 |  | 0.860 | 0.902 | 0.433 | 0.019 |
|  |  | **SWITZERLAND** | |  |  |  |  |  |  |  |
| Low | Inactive | 0.209 | 0.228 | 0.674 | 0.968 |  | 0.457 | 0.508 | 0.882 | 0.991 |
|  | Active | 0.791 | 0.773 | 0.326 | 0.032 |  | 0.543 | 0.492 | 0.118 | 0.009 |
| Middle | Inactive | 0.053 | 0.109 | 0.600 | 0.970 |  | 0.389 | 0.380 | 0.827 | 0.989 |
|  | Active | 0.947 | 0.891 | 0.400 | 0.030 |  | 0.611 | 0.620 | 0.173 | 0.011 |
| High | Inactive | 0.034 | 0.081 | 0.468 | 0.931 |  | 0.235 | 0.262 | 0.710 | 0.971 |
|  | Active | 0.966 | 0.919 | 0.532 | 0.069 |  | 0.765 | 0.738 | 0.290 | 0.029 |
|  |  | **TURIN** |  |  |  |  |  |  |  |  |
| Low | Inactive | 0.134 | 0.349 | 0.857 | 0.989 |  | 0.433 | 0.617 | 0.944 | 0.997 |
|  | Active | 0.866 | 0.652 | 0.143 | 0.011 |  | 0.567 | 0.383 | 0.056 | 0.003 |
| Middle | Inactive | 0.065 | 0.213 | 0.727 | 0.969 |  | 0.208 | 0.400 | 0.892 | 0.994 |
|  | Active | 0.935 | 0.788 | 0.273 | 0.031 |  | 0.792 | 0.600 | 0.108 | 0.006 |
| High | Inactive | 0.047 | 0.141 | 0.495 | 0.860 |  | 0.134 | 0.285 | 0.772 | 0.976 |
|  | Active | 0.953 | 0.859 | 0.506 | 0.140 |  | 0.866 | 0.715 | 0.228 | 0.024 |
|  |  | **TUSCANY** | |  |  |  |  |  |  |  |
| Low | Inactive | 0.103 | 0.316 | 0.788 | 0.970 |  | 0.418 | 0.617 | 0.931 | 0.994 |
|  | Active | 0.897 | 0.685 | 0.212 | 0.030 |  | 0.582 | 0.383 | 0.069 | 0.006 |
| Middle | Inactive | 0.067 | 0.166 | 0.638 | 0.955 |  | 0.232 | 0.389 | 0.864 | 0.991 |
|  | Active | 0.933 | 0.834 | 0.362 | 0.045 |  | 0.768 | 0.611 | 0.136 | 0.009 |
| High | Inactive | 0.060 | 0.108 | 0.408 | 0.847 |  | 0.156 | 0.239 | 0.767 | 0.985 |
|  | Active | 0.940 | 0.892 | 0.592 | 0.153 |  | 0.844 | 0.761 | 0.233 | 0.015 |

Self-reported economic activity classified as ‘inactive’ (not seeking work, student, housewife) or ‘active’ (paid work, seeking work). Unemployed were classified among inactive in Norway, Sweden, Switzerland, and Madrid.

| Table S11  Potential reduction (in %) of relative educational inequalities in all-cause mortality between low and high educated, upward levelling scenario, by risk factor, country and sex | | | | | | | | | | | | | |
| --- | --- | --- | --- | --- | --- | --- | --- | --- | --- | --- | --- | --- | --- |
|  | Lifestyle risk factors | | | | | | Social and economic risk factors | | | | | |  |
|  | Smoking | | Over-weight | | Physical  inactivity | | Social participation | | Low income | | Economic inactivity | |  |
| Population | M | W | M | W | M | W | M | W | M | W | M | W |  |
| Finland | 17 | 8 | 2 | 8 | 2 | 1 | 3 | 9 | 20 | 10 | 19 | 17 |  |
| Sweden | 18 | 11 | 6 | 9 | na | na | 4 | 10 | 20 | 8 | 14* | 15* |  |
| Norway | 26 | 18 | 6 | 8 | 4 | 5 | 6 | 9 | 17 | 12 | 20* | 18* |  |
| Denmark | 19 | 9 | 9 | 10 | 6 | 11 | 11 | 9 | 17 | 10 | na | na |  |
| England &W | 26 | 16 | 6 | 12 | na | na | 10 | 11 | 21 | 14 | 14 | 16 |  |
| Scotland | 22 | 20 | 5 | 4 | na | na | na | na | 20 | 11 | na | na |  |
| Netherlands | 13 | 7 | 8 | 12 | 2 | 9 | 5 | 9 | 20 | 13 | na | na |  |
| Belgium | 8 | 2 | 8 | 15 | 6 | 19 | 9 | 4 | 20 | 13 | na | na |  |
| France | 6 | 2 | 9 | 16 | na | na | 8 | 16 | 18 | 17 | na | na |  |
| Switzerland | 9 | 2 | 8 | 18 | 8 | 17 | na | na | 18 | 14 | 19* | 18* |  |
| Austria | 11 | 2 | 12 | 20 | na | na | 6 | 21 | na | na | 17 | 27 |  |
| Barcelona | 8 | 2 | 8 | 25 | 8 | 11 | 14 | 8 | na | na | na | na |  |
| Basque C | 9 | 4 | 5 | 34 | 9 | 17 | 17 | 13 | na | na | 18 | 82 |  |
| Madrid | 8 | 2 | 9 | 29 | 9 | 13 | 17 | 9 | na | na | 19* | 40* |  |
| Turin | 5 | 2 | 10 | 42 | 3 | 16 | 9 | 11 | na | na | 25* | 57* |  |
| Tuscany | 4 | 1 | 9 | 24 | 3 | 9 | 8 | 7 | na | na | na | na |  |
| Hungary | na | na | 2 | 11 | na | na | 8 | 5 | 16 | 15 | 25 | 31 |  |
| Czech R | 19 | 5 | 6 | 10 | 2 | 3 | na | na | 12 | 8 | na | na |  |
| Poland | 21 | 5 | 1 | 12 | na | na | 8 | 4 | 20 | 10 | na | na |  |
| Lithuania | 14 | 3 | 2 | 4 | 6 | 13 | na | na | na | na | na | na |  |
| Estonia | 14 | 6 | 0 | 10 | 3 | 5 | na | na | na | na | na | na |  |
| All ^#^ | 14 | 5 | 6 | 12 | 4 | 11 | 8 | 9 | 18 | 11 | 18/19* | 27/18* |  |
| Notes:* economically inactive include unemployed . Na: not available. # European average (arithmetic mean). M=men, W=women. Yellow: reduction of educational inequalities in all-cause mortality by 0 – 5 %. Light green: reduction of educational inequalities in all-cause mortality by 6 – 19 %. Green: reduction of educational inequalities in all-cause mortality by at least 20 %. | | | | | | | | | | | | | |

| **Table S12**  **Potential reduction (in %) of relative educational inequalities in all-cause mortality between low and high educated, best practice scenario, by risk factor, country and sex.** | | | | | | | | | | | | | |
| --- | --- | --- | --- | --- | --- | --- | --- | --- | --- | --- | --- | --- | --- |
|  | Lifestyle risk factors | | | | | | Social and economic risk factors | | | | | |  |
|  | Smoking | | Over-weight | | Physical  inactivity | | Social participation | | Low income | | Economic inactivity | |  |
| Population | M | W | M | W | M | W | M | W | M | W | M | W |  |
| Finland | 8 | 7 | **ref** | 0 | **ref** | **ref** | -7 | 2 | 3 | 0 | 2 | **ref** |  |
| Sweden | 8 | 23 | 0 | 0 | na | na | -5 | 0 | 3 | -1 | **ref*** | **ref*** |  |
| Norway | 14 | 26 | 0 | 0 | 4 | 5 | -3 | 0 | 4 | 3 | 2* | 4* |  |
| Denmark | 11 | 25 | 1 | 0 | 3 | 7 | 1 | 0 | 1 | -2 | na | na |  |
| England &W | 15 | 32 | 7 | 7 | na | na | 1 | 1 | 2 | 2 | **ref** | 0 |  |
| Scotland | 15 | 32 | 3 | **ref** | na | na | na | na | 3 | 1 | na | na |  |
| Netherlands | 9 | 25 | 0 | 0 | 3 | 11 | **ref** | 0 | 3 | 1 | na | na |  |
| Belgium | 2 | 15 | 0 | 0 | 5 | 17 | 1 | **ref** | 2 | 1 | na | na |  |
| France | **ref** | 3 | 1 | 0 | na | na | 3 | 4 | 2 | 4 | na | na |  |
| Switzerland | 4 | 12 | 0 | 0 | 3 | 10 | na | na | 3 | 2 | 0* | -1* |  |
| Austria | -1 | 2 | 2 | 0 | na | na | 0 | 6 | na | na | 1 | 9 |  |
| Barcelona | -4 | -5 | 3 | 3 | 7 | 14 | 8 | 1 | na | na | na | na |  |
| Basque C | -1 | 4 | 1 | 0 | 9 | 21 | 10 | 2 | na | na | 1 | 46 |  |
| Madrid | -5 | -7 | 3 | 3 | 9 | 19 | 10 | 1 | na | na | 0* | 10 |  |
| Turin | -2 | **ref** | 0 | 0 | 4 | 20 | 3 | -7 | na | na | 9* | 11 |  |
| Tuscany | -2 |  | 0 | 0 | 3 | 12 | 3 | -4 | na | na | na | na |  |
| Hungary | na | na | 1 | 2 | na | na | 5 | 1 | 1 | 2 | 14 | 19 |  |
| Czech R | 10 | 7 | 2 | 4 | 3 | 5 | na | na | **ref** | **ref** | na | na |  |
| Poland | 16 | 9 | 0 | 3 | na | na | 5 | 1 | 1 | 1 | na | na |  |
| Lithuania | 9 | 0 | -2 | -6 | 6 | 17 | na | na | na | na | na | na |  |
| Estonia | 4 | 6 | -1 | 4 | -3 | 7 | na | na | na | na | na | na |  |
| All ^#^ | 9 | 12 | 1 | 0 | 4 | 11 | 1 | 1 | 2 | 1 | 2/1* | 19/7* |  |
| Notes:Na: not available  # Europe (arithmetic mean), M=men, W=women  * economically inactive include unemployed (England / Wales and Finland is reference country for men and women respectively). Sweden is reference of countries with unemployed included in the active, both among men and women. Ref: Country serving as reference for best practice Yellow: reduction of educational inequalities in all-cause mortality by 5 % or less. Light green: reduction of educational inequalities in all-cause mortality by 6 – 19 %. Green: reduction of educational inequalities in all-cause mortality by at least 20 % | | | | | | | | | | | | | |
